# Interactions between the metabolic reprogramming of liver cancer and tumor microenvironment

**DOI:** 10.3389/fimmu.2025.1494788

**Published:** 2025-02-14

**Authors:** Haoqiang Yang, Jinghui Li, Yiting Niu, Tao Zhou, Pengyu Zhang, Yang Liu, Yanjun Li

**Affiliations:** ^1^ Third Hospital of Shanxi Medical University, Shanxi Bethune Hospital, Shanxi Academy of Medical Sciences, Tongji Shanxi Hospital, Taiyuan, China; ^2^ Department of Hepatobiliary Surgery, Shanxi Bethune Hospital, Shanxi Academy of Medical Sciences, TongjiShanxi Hospital, Third Hospital of Shanxi Medical University, Taiyuan, Shanxi, China

**Keywords:** hepatocellular carcinoma, tumor microenvironment, metabolism reprogram, signaling pathways, immune evasion

## Abstract

Metabolic reprogramming is one of the major biological features of malignant tumors, playing a crucial role in the initiation and progression of cancer. The tumor microenvironment consists of various non-cancer cells, such as hepatic stellate cells, cancer-associated fibroblasts (CAFs), immune cells, as well as extracellular matrix and soluble substances. In liver cancer, metabolic reprogramming not only affects its own growth and survival but also interacts with other non-cancer cells by influencing the expression and release of metabolites and cytokines (such as lactate, PGE2, arginine). This interaction leads to acidification of the microenvironment and restricts the uptake of nutrients by other non-cancer cells, resulting in metabolic competition and symbiosis. At the same time, metabolic reprogramming in neighboring cells during proliferation and differentiation processes also impacts tumor immunity. This article provides a comprehensive overview of the metabolic crosstalk between liver cancer cells and their tumor microenvironment, deepening our understanding of relevant findings and pathways. This contributes to further understanding the regulation of cancer development and immune evasion mechanisms while providing assistance in advancing personalized therapies targeting metabolic pathways for anti-cancer treatment.

## 1 Introduction

The tumor microenvironment (TME) is a complex and evolving entity The composition of TME varies depending on the type of tumor, but its hallmark features include immune cells, stromal cells, blood vessels, and extracellular matrix (ECM) (1). During the process of tumor development, cancer cells can interact with various components of TME by secreting various cytokines, chemokines, and other factors. They tend to reprogram surrounding cells and shape their microenvironment to support the progression, local invasion, and metastatic spread of cancer cells. For example, elevated Zinc finger protein 64 (ZFP64) in liver cancer promotes an immunosuppressive tumor microenvironment to achieve resistance to anti-PD-1 therapy (2). The TME in liver cancer not only exerts influence on the tumor cells, but also orchestrates their immune evasion through diverse pathways. Currently, scholars have partially elucidated the mechanisms underlying the impact of spatial alterations in the liver cancer microenvironment on its therapeutic response. For instance, Liu Yao et al. conducted a study investigating the spatial structure of the liver cancer microenvironment and revealed that interactions between CAFs and SPP1^+^ macrophages facilitate ECM accumulation through ligand-receptor interactions, thereby influencing the efficacy of immunotherapy for liver cancer (3). Numerous studies have demonstrated that the tumor microenvironment plays a crucial role in the occurrence, development, treatment response and prognosis of liver cancer. These findings underscore the significance of the microenvironment in both the prognosis and treatment of liver cancer, necessitating a more comprehensive comprehension of cellular interactions within the tumor microenvironment (TME).

In 2020, primary liver cancer emerged as the sixth most prevalent cancer globally and the third most fatal (4). Estimates suggest that over one million people will be affected by liver cancer annually by 2025 (5). Liver cancer cells proliferate in a rapid and uncontrolled manner, often existing in an environment of hypoxia and nutrient deficiency. Therefore, they must undergo adaptive metabolic remodeling to meet their high energy demands and create a favorable microenvironment for growth. This phenomenon is known as “metabolic reprogramming”. The glucose metabolism pathway of liver cancer cells undergoes significant changes, with glycolysis and lactate fermentation occurring at a high rate even under different oxygen levels. This phenomenon is referred to as the Warburg effect (6). In addition to the reshaping of sugar metabolism, metabolic reprogramming also encompasses crucial processes such as amino acid and lipid synthesis and decomposition. Through these adaptive metabolic alterations, liver cancer cells efficiently fulfill their energy demands. Moreover, metabolic reprogramming can confer resistance to cancer treatment by impacting programmed cell death and associated events in liver cancer cells. For instance, the upregulation of glucose metabolism and phospholipid synthesis has been shown to enhance the radio resistance of hepatocellular carcinoma (HCC) cells through modulation of cytochrome c, indicating a poor prognosis for response to radiation therapy (7). Recent studies have demonstrated that metabolic reprogramming occurs not only in tumor cells, but also induces diverse forms of metabolic alterations, such as aerobic glycolysis, in non-cancerous cells within the tumor microenvironment (TME). These adaptations enable tumor cells to effectively respond to changes in nutrient availability, thereby facilitating their proliferation and growth (8). Meanwhile, these changes also induce immune cells to exhibit intricate metabolic patterns, which not only impact the activation state of immune cells but also exert significant influences on their differentiation process, thereby eliciting tumor immune evasion (9). Revised sentence: “For instance, the deficiency of RIPK3 in tumor-associated macrophages (TAMs) results in PPAR activation, thereby promoting fatty acid metabolism and inducing M2-like TAM polarization, which ultimately enhances immune suppression activity in liver cancer (10).

This review aims to investigate the metabolic interactions between cancerous and non-cancerous cells within the microenvironment of liver cancer. The initial section of this article will comprehensively present the metabolic reprogramming of liver cancer, with a specific focus on its fundamental processes and latest clinical advancements. Subsequently, an overview of the tumor microenvironment (TME) in liver cancer will be provided, accompanied by a detailed discussion on the metabolic interplay between liver cancer cells and other cell types within the TME, elucidating their biological significance. This study not only contributes to unraveling the mechanisms underlying the occurrence and progression of liver cancer but also enhances immunotherapy efficacy by targeting metabolic processes to improve immune microenvironment”.

## 2 Metabolic reprogramming in hepatocellular carcinoma.

### 2.1 Glucose metabolism

**Figure 1:**
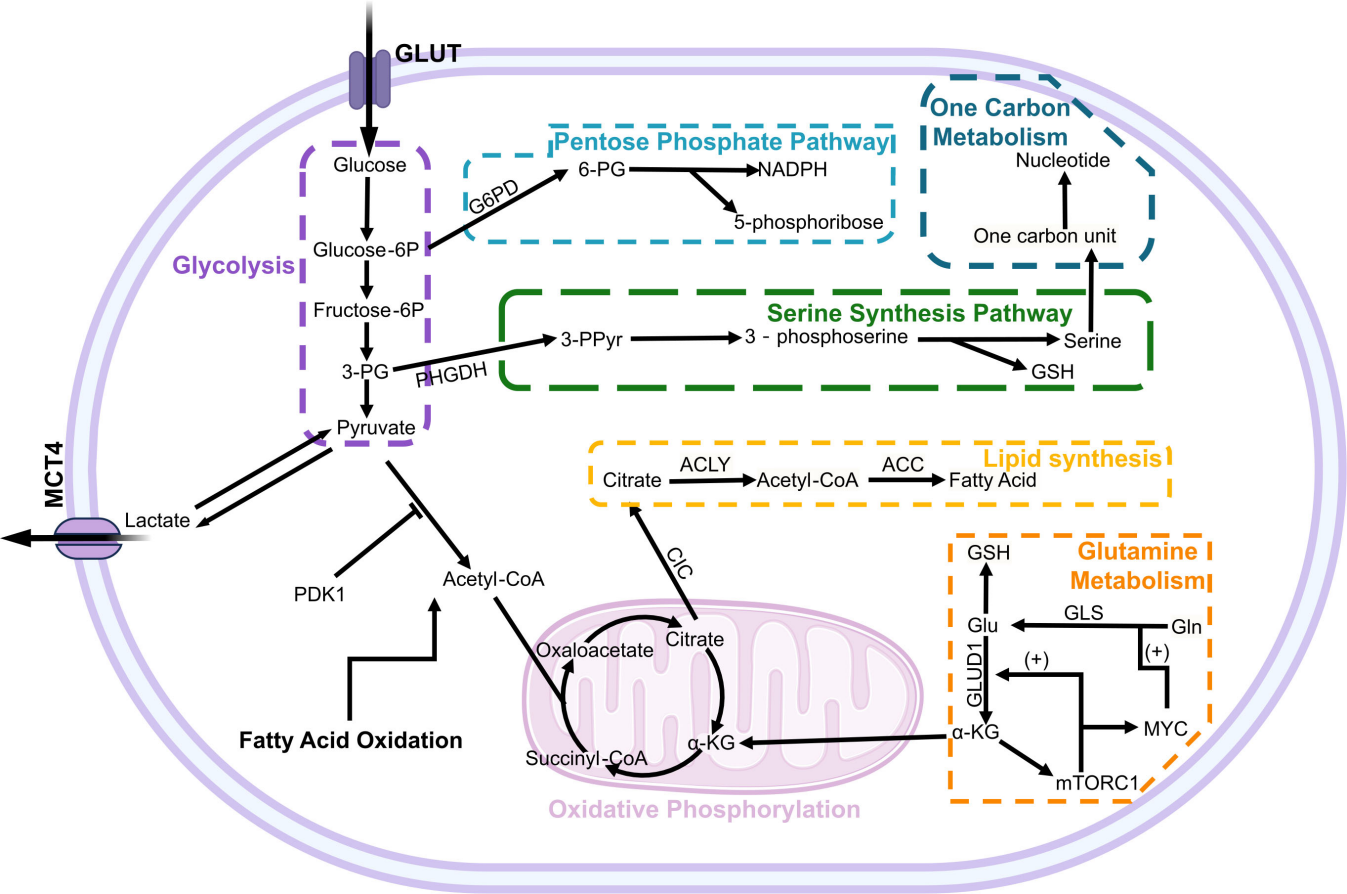
The metabolic pathways and regulation in hepatocellular carcinoma cells. The metabolism of glucose, glutamine, and fatty acids is intricately regulated by a multitude of factors, including the signaling pathways associated with cancer and tumor suppression. In glucose metabolism, PDK1 exerts inhibitory effects on key enzymes involved in the pyruvate dehydrogenase reaction to modulate the aerobic respiration pathway. Within glutamine metabolism, mTORC1 activation by α-KG establishes a positive feedback loop that enhances GLUD1 and MYC-GLS expression to promote glutamine utilization. Glucose-6P, glucose 6-phosphate; Fructose-6P, fructose-6-phosphate; 3-PG, 3-phosphoglyceric acid; PHGDH, phosphoglycerate dehydrogenase; 3-PPyr, 3-phosphohydroxypyruvate; 6-PG, 6-phosphogluconic acid; GSH, glutathione; ACLY, ATP-citrate lyase; ACC, Acetyl CoA carboxylase; GLUD1, glutamate dehydrogenase 1; GLS, Glutaminase; Gln, Glutamine; CIC, Citrate carrier.

The normal cellular energy metabolism process begins with the acquisition of energy and pyruvic acid through glycolysis in the cytoplasm under aerobic conditions, followed by oxidative phosphorylation of pyruvic acid to acetyl-CoA in the mitochondria, entering the tricarboxylic acid cycle (TCA cycle), or conversion of pyruvic acid to lactic acid in the cytoplasm under anaerobic conditions (11). Unlike normal cells, cancer cells tend to undergo glycolysis metabolism with high lactate production even under aerobic conditions, rather than aerobic respiration, known as the Warburg effect. For instance, Pyruvate dehydrogenase kinase 1 (PDK1) can inhibit the tricarboxylic acid (TCA) cycle by phosphorylating the pyruvate dehydrogenase complex (PDC) in a manner that is independent of hypoxic conditions (12, 13) (**Figure 1**). Current research indicates that HK2, HIF-1, PDK1 are all driving factors for cells to undergo aerobic glycolysis related targets. The study suggests that HK2 is typically the sole expressed hexokinase in hepatocellular carcinoma (HCC) cells. Low-dose inhibitors of HK2 can effectively target HCC cells, and when combined with metformin or sorafenib, they significantly enhance apoptosis and inhibit tumor growth in human HCC cells (14). Normal cells obtain a higher ATP yield through aerobic respiration, while cancer cells produce ATP at a faster rate through glycolytic respiration, giving them a competitive advantage in obtaining more energy resources (15). In the aforementioned process, GLUT1 serves as the primary transmembrane transport protein responsible for establishing a glucose uptake advantage. Numerous studies have revealed a significant upregulation of GLUT1 expression in liver cancer tissues, which is positively correlated with tumor size. Patients exhibiting high levels of GLUT1 expression demonstrate lower overall survival (OS) rates and recurrence-free survival (RFS) (16). However, researchers found that although the expression of GLUT2 (SLC2A2) was significantly lower in liver cancer than that of GLUT1, GLUT2 had a more significant prognostic value, with the late clinical staging and overall survival of HCC patients highly dependent on GLUT2 expression (17). Repression of GLUT1 expression additionally leads to a decrease in glucose uptake and lactate secretion, consequently resulting in reduced proliferation and migration capacity of HCC cells (18). However, recent studies have demonstrated that the regulation of glucose uptake in liver cancer cells involves a collaborative interplay between Sodium glucose cotransporter 2 (SGLT2) and glucose transporter 1 (GLUT1) (19). Canagliflozin (Sodium glucose cotransporter 2 inhibitor) directly slows down the progression of liver cancer by inhibiting glycolysis and angiogenesis. Both SGLT2 and GLUT1 support the high metabolic rate of glycolysis in cancer cells, while also competing with glucose in the tumor microenvironment to restrict uptake by surrounding cells (20). For example during T cell activation period blocking glycolysis or limiting glucose uptake will reduce cytokine (such as IFN-γ) production and impact CD8^+^ T cell response (21). Research findings have demonstrated that PRMT3 facilitates glycolysis by methylating LDHA, thereby augmenting LDHA activity and fostering HCC proliferation (22). At the same time, large amounts of lactate generated by cancer cells through aerobic glycolysis mediate AMPK-SCD1-enhanced resistance of liver cancer cells to ferritin damage (23). Some lactate is transported outwards leading to environmental acidosis and cytotoxicity formation.

The partial 3-phosphoglycerate produced by glycolysis is converted into 3-phosphohydroxypyruvate as a precursor for the serine synthesis pathway (SSP) (24). Under conditions of nutrient deprivation, activation of SSP increases the production of glutathione (GSH), cell cycle progression, and nucleic acid synthesis to maintain basic cellular survival and proliferation (25). The rate-limiting enzyme of the serine synthesis pathway (SSP) is phosphoglycerate dehydrogenase (PHGDH), which exhibits pronounced upregulation in HCC and demonstrates a close correlation with its pathogenesis and proliferation (26). Concurrently, ZEB1 amplifies SSP by transcriptionally activating PHGDH, thereby exacerbating tumorigenesis and metastasis (27). In addition to the SSP pathway, glucose also participates in the pentose phosphate pathway (PPP).The PPP serves as an alternative metabolic pathway for glucose in the cytoplasm, generating NADPH and 5-phosphoribose. Among these products, 5-phosphoribose is one of the components for DNA synthesis. Rapid division of cancer cells requires a large amount of DNA and its precursors, thus activation of the PPP meets the material demands for rapid cell division in cancer cells (28). In the accelerated metabolism of cancer cells, high levels of reactive oxygen species (ROS) are generated. Cancer cells are susceptible to oxidative stress-induced cell death; however, NADPH produced by the PPP plays a crucial role in reducing biosynthesis, protecting cells from ROS damage (29). Previous studies have demonstrated that ID1 confers chemoresistance to HCC cells against oxaliplatin by modulating the activation of the G6PD pathway. Moreover, elevated expression levels of both ID1 and G6PD are significantly associated with an unfavorable prognosis in liver cancer (30).

### 2.2 Glutamine metabolism

Glutamine (Gln) is the most abundant free amino acid in blood and a major physiological source of nitrogen in mammalian cells. Similar to the glycolysis mentioned above, extracellular glutamine is transported by the alanine-serine-cysteine transporter 2 (ASCT2), which is highly expressed in various cancers, not only limited to liver cancer but also including breast cancer and lung cancer (31). Similar to GLUT1 mentioned above, patients with high expression of ASCT2 have a significantly lower survival expectancy than those with low expression of ASCT2 (16). Subsequently, glutamine undergoes catalysis by glutaminase (GLS) to be converted into glutamate within the mitochondria (32). In the aforementioned metabolic process, particular attention should be given to glutaminase (GLS), which encompasses two subtypes referred to as GLS1 (kidney-type glutaminases) and GLS2 (liver-type glutaminases). Revised sentence: Previous studies have demonstrated a robust upregulation of GLS1 in hepatocellular carcinoma (HCC), establishing it as a prominent prognostic biomarker. Therapeutic interventions targeting GLS1, such as canagliflozin, exhibit the potential to impede glutamine metabolism, induce apoptosis in malignant cells, and sensitize HCC towards cisplatin(CPT) (33, 34). In contrast to GLS1, the expression of GLS2 is predominantly confined to non-tumor hepatic cells. Upregulation of GLS2 in liver cancer cells primarily occurs within mitochondria and elicits an anti-proliferative response by arresting the cell cycle at G2/M phase. Moreover, it enhances sensitivity to ionizing radiation (33, 35, 36). However, GLS2 exhibits tumor heterogeneity and is found to be upregulated in specific malignancies, such as breast cancer, thereby promoting oncogenesis (37). Researchers have discovered that glutamate, the hydrolysis product of glutamine, undergoes metabolism and degradation within liver cancer mitochondria to generate ATP (38). This is mainly due to the Warburg effect and the maintenance of fatty acid metabolism, citric acid and pyruvic acid cannot replenish the TCA cycle inside mitochondria, leading to TCA cycle disruption. Subsequently, glutamate is further deaminated by glutamate dehydrogenase (GDH) to generate α-ketoglutarate (α-KG), which serves as an intermediate of the TCA cycle, rebuilding the TCA cycle in cancer cells (25). The GDH comprises two subtypes, with hGLUD1 encoded by the GLUD1 gene demonstrating the highest expression in hepatic tissue. It exhibits significant upregulation in both hepatocellular carcinoma (HCC) human samples and HepG2 cells (39). The research findings demonstrate that the silencing of GLUD1 exerts an impact on redox homeostasis, thereby inducing mitochondrial apoptosis in HepG2 cells; however, no significant influence on the proliferation of normal hepatic cells was observed (40). However, the study by You et al. found that shRNA-mediated silencing of GLUD1 actually enhances the growth and migration of HepG2 and Huh7 cells. Therefore, further research is needed to investigate GLUD1 and its glutamine metabolism mechanism in order to evaluate its therapeutic value as a target for metabolic treatment (41). In addition to rebuilding the TCA cycle and antioxidant effects, as a carbon and nitrogen source, glutamine promotes synthesis of macromolecules such as fatty acids, proteins and nucleotides. Furthermore, it influences cytokines such as tumor necrosis factor-α (TNF-α) and interleukin-6 (IL-6), enhancing activity of other factors in the immune system (42). However, current research has not yet fully elucidated the interrelationship between glutamine metabolism and glucose metabolism in hepatocellular carcinoma. Nonetheless, existing studies have highlighted the complexity and significance of this relationship. For example, Zhou et al. demonstrated that variations in glucose concentration do not impair the role of glutamine in sustaining cell growth, but different glucose levels can activate distinct glutamine catabolic pathways (43). These findings underscore the need for further investigation into the mechanisms governing the triggering and transformation of metabolic pathways.

Multiple genes are involved in the reprogramming of glutamine metabolism, such as MYC, P53, and glutamate dehydrogenase (GDH) (44). MYC binds to the promoter of glutamine metabolism genes, directly stimulating glutamine metabolism. It can also indirectly stimulate glutamine metabolism by inhibiting the expression of miR-23a/b, an inhibitor of the glutaminase isozyme (GLS1) (45). Meanwhile, α-KG generated from glutamine activates mTORC1 and inhibits autophagy. mTORC1 enhances glutamine decomposition by activating MYC-GLS and GLUD1, establishing a positive feedback loop that may be responsible for substantial glutamine consumption (46) (**Figure 1**). In summary, glutamine is crucial for cell survival as it not only involves TCA cycle reconstruction but also participates in protein, lipid, nucleotide metabolism and plays relevant roles in signaling pathways. Therefore, previous researchers have employed the expression levels of 41 genes associated with glutamine metabolism to establish Glutamine Metabolism Scoring (GMScore) for assessing glutamine metabolism activity and predicting the prognosis of HCC, as well as the response to immune checkpoint inhibitor (ICI) therapy (47). Meanwhile, with regard to the glutamine-related metabolic pathways, current methods can use amino acid depletion therapy to reduce the intracellular Gln level to a level that does not allow cell proliferation. For example, researchers have used crisantaspase and glutamine synthetase inhibitor methionine-L-sulfoximine (MSO) to deplete glutamine, which significantly impairs the growth of human hepatocellular carcinoma xenografts (48).

### 2.3 Lipid metabolism

Numerous studies have consistently demonstrated that a prominent hallmark of cancer cell metabolism involves the remodeling of lipid metabolism, encompassing fatty acid transportation, *de novo* lipid synthesis, storage within lipid droplets, and β-oxidation. This intricate process is intricately linked to the upregulation of fatty acid *de novo* synthesis and associated enzyme expression in malignant tumors, thereby conferring growth advantages upon tumor cells (49–51). Lipid metabolism includes both fatty acid synthesis and fatty acid oxidation (such as β-oxidation), and an imbalance between them can lead to lipid accumulation. In mammalian cells, FAs can be obtained directly from the surrounding microenvironment by exogenous intake, or synthesized *de novo* using nutrients such as glucose or glutamine. However, cancer cells exhibit a heightened reliance on *de novo* fatty acid synthesis to sustain their lipid metabolic equilibrium and establish diverse functional lipid reservoirs, which are essential for the initiation, survival, and progression of HCC(Hepatocellular carcinoma) (52). For instance, stearoyl-CoA desaturase 1 (SCD1) utilizes the mechanical signaling pathway to orchestrate lipid metabolism reprogramming and enhance membrane fluidity, thereby facilitating invasive migration and metastasis of hepatocellular carcinoma (HCC) cells (53). It is noteworthy that the observed lipid reprogramming phenomena are not limited to cancer, as they have also been observed in murine models of liver regeneration and direct liver hyperplasia (54). The discovery suggests a robust association between lipid metabolism and the progression of NASH-cirrhosis, a preliminary stage preceding HCC. Therefore, lipid synthesis is closely related to cell growth and is also a prerequisite for cell division (55).

Research suggests that the transition from lipid uptake to *de novo* lipogenesis in cancer cells leads to an increase in membrane lipid saturation, resulting in higher levels of saturated and monounsaturated phospholipids, thereby reducing lipid peroxidation potential to potentially protect cancer cells from oxidative damage (56). In *de novo* fatty acid (FA) synthesis, the citrate carrier (CIC/SLC25A1) transports TCA cycle product citrate across the mitochondrial inner membrane into the cytoplasm to participate in metabolism (25). The study revealed that parthenolide (PTL) induces cell cycle arrest at the G1 phase and promotes apoptosis by inhibiting SLC25A1, thereby diminishing the stemness of liver cancer stem cells (LCSCs) (57). The growth of liver cancer *in vivo* was effectively suppressed by PTL, demonstrating no significant observed toxic reactions. Meanwhile, as a specific inhibitor of SLC25A1, CTPI-2 significantly mitigates lipid metabolism disorders in non-alcoholic steatohepatitis (NASH), thereby impeding its progression to hepatic steatosis and reducing inflammatory macrophage infiltration in the liver and adipose tissue (58). The ATP citrate lyase (ACLY) serves as the initial rate-limiting enzyme in *de novo* lipogenesis, exhibiting high expression levels in both hepatocellular carcinoma (HCC) tissues and liver cancer stem cells (LTICs). This significant correlation with unfavorable prognosis, disease progression, and metastasis among HCC patients remains evident (59). The study revealed that sorafenib-resistant liver cancer cells exhibit active lipid metabolism and display elevated expression levels of ACLY (60). Gene Knockdown targeting ACLY not only exhibited potent inhibitory effects on the proliferation, migration and invasion of HCC cells, but also effectively overcame sorafenib resistance, particularly under hypoxic conditions (59, 60).

In addition to *de novo* synthesis of fatty acids, the uptake and clearance of extracellular FAs also play an important role in maintaining the lipid requirements of cancer cells under hypoxic and metabolic stress conditions (51). Specialized transport proteins are required to facilitate efficient movement of FAs across the cell membrane during the process of exogenous FA uptake. Among them, fatty acid translocase (CD36, FAT), fatty acid transport protein family [FATPs, solute carrier protein family 27 (SLC27)] and plasma membrane fatty acid binding protein (FABPPM) all exhibit high levels of gene and protein expression in tumors (61). Previous studies have indicated that CD36 enhances the uptake of fatty acids (FAs) in HCC by promoting the expression of AKR1C2. Knocking down CD36 inhibits the uptake of FAs, thus suppressing the proliferation and metastasis of HCC (62). Accumulation of FAs and neutral lipids in non-adipose tissue is known to rapidly stimulate apoptosis (63). Exogenous FAs are stored in lipid droplets (LDs) after intake, chelating excess FAs in the form of TAG and sterol esters to avoid cell damage. Therefore, in cancer cells, LDs coordinate lipid transport consumption, chelate toxic lipids, and avoid lipid toxicity-induced cell damage and other biological functions, suggesting that LDs participate in the enhancement of cancer cell adaptability and stress resistance (64). Dihydroartemisinin(DHA) reduces lipid droplet accumulation in hepatocellular carcinoma by inhibiting YAP1 and enhances sensitivity to PD-1 therapy (65).

In the progression of tumors, lipid biosynthesis, as part of cancer cell synthesis metabolism, not only provides energy for rapidly proliferating cancer cells, but also plays an important role in cell transformation and cancer development by serving as signaling molecules (such as phosphoinositides, phospholipids, sphingolipids) to mediate cellular signal transduction and participate in post-translational modification of proteins (such as protein S-acylation) (50).

### 2.4 Amino acid metabolism

Amino acids are the building blocks of proteins and serve as raw materials for intracellular protein synthesis. Amino acid metabolism, one of the body’s three major metabolic processes, includes pathways like glutamine, serine, and glycine metabolism. In this section, we focus on the distinct nutritional requirements and biosynthetic processes of cancer cells compared to normal cells. Some amino acids, such as asparagine, arginine, methionine, cysteine, and branched-chain amino acids, have acquired new biological functions.

The crucial role of asparagine in the survival mechanism of cancer cells, and Pavlova et al. found that when asparagine is restricted, the biosynthesis of aspartate becomes the basis for tumor cell mitochondrial respiration (66). Unlike other amino acids, aspartate is not involved in catabolism but rather serves as an amino acid exchange factor for retrograde transport to absorb essential amino acids and balance intracellular or extracellular levels of other amino acids including glycine, histidine, serine, and threonine (67). Research has shown that upregulation of PTTG1 expression promotes transcriptional activation of asparagine synthetase (ASNS), a key activator of Asn metabolism, enhancing Asn metabolism and activating mTOR pathway to promote progression of hepatocellular carcinoma (68).

Arginine (Arg) is an important component of the ornithine cycle, and arginine succinate synthase 1 (ASS1) and arginine succinate lyase (ASL) are involved in the synthesis of Arg from citrulline through two-step reactions, participating in the formation of growth hormone and urea (69). Unlike normal cells, most tumor cells lack the rate-limiting enzyme for Arg synthesis, arginine succinate synthetase (ASS), showing a strong dependence on Arg (70). To maintain high levels of Arg and its metabolism in hepatocellular carcinoma cells, RBM39 promotes the synthesis of asparagine synthetase (ASNS) to enhance the absorption of asparagine for the formation of a positive feedback loop (71).

Methionine serves as an essential amino acid and is a precursor to succinyl-CoA, S-adenosyl methionine (SAM), cysteine, among other products. SAM interferes with signal transduction mechanisms at different levels and largely contributes to hepatic carcinogenesis and tumorigenesis mechanisms (72). In liver cells, methionine is converted into S-adenosyl methionine (SAMe) by methionine adenosyl transferase. The researchers discovered that the majority of liver cancer cells undergo cell cycle arrest and suffer from severe DNA damage when subjected to acute methionine deprivation (73). Even if these cancer cells possess the ability to synthesize normal or excessive quantities of methionine, they still rely on exogenous methionine for sustenance (74). This reliance has emerged as a significant hallmark of cancer metabolism, referred to as the ‘Hoffman effect’, and is also acknowledged as an addiction to methionine. The Hoffman effect is considered even more significant than Warburg effect because it is the metabolic change that occurs in all known cancer cells.

Cysteine is an essential component of all proteins, serving not only as a precursor for important metabolites such as the antioxidant GSH, coenzyme A, sulfates, and taurine, but also as a limiting amino acid in GSH synthesis (75). Inability of cancer cells to acquire sufficient cysteine from the extracellular environment leads to an inability to meet their own antioxidant demands, ultimately resulting in lethal oxidative stress. For example, cysteine starvation can induce ferroptosis by downregulating GSH levels (76). Research on hepatocellular carcinoma has shown that the RNA-binding protein partner of NOB1 (PNO1) mediates an increase in intracellular glutamine levels, activating the Cystine/Glutamate Antiporter System x_c_^−^ to import additional cysteine and ultimately upregulate GSH biosynthesis to counteract ferroptosis (77).

Branched-chain amino acids (BCAAs), including leucine, isoleucine, and valine, belong to a group of essential amino acids. The metabolic reprogramming of BCAAs involves changes in the expression and activity of BCAA transport proteins and metabolic enzymes. For example, the decreased methylation level of the BCAT1 promoter in HCC tissues leads to increased BCAT1 expression, over activating the AKT signaling pathway and promoting epithelial-to-mesenchymal transition (EMT), thereby facilitating the occurrence and metastasis of liver cancer cells (78, 79).

In the occurrence, progression, and prognosis of tumors, the above-mentioned amino acids have preliminarily demonstrated the complexity and significance of local amino acid metabolism in tumors. Concurrently, based on the differential requirements for amino acids between normal cells and tumor cells, various treatment methods have been proposed such as amino acid depletion therapy, inhibition of amino acid transporters, and suppression of amino acid biogenesis. For example, missaen et al. found that deprivation of arginine deprives the involvement in integrated stress response (ISR), thereby promoting HCC cell cycle arrest and stasis, ultimately leading to cell apoptosis (80).

### 2.5 Hepatocellular carcinoma metabolic heterogeneity and its diagnostic value

The existence of intra-tumor heterogeneity (ITH) has been firmly established in numerous cancers, encompassing temporal, spatial, and TME (metabolic and immunological components) heterogeneity, and further exerting influences on the heterogeneous behavior of tumor cells and alterations in the immune cell immune response (81). Concerning the temporal heterogeneity of liver cancer, its most prominent feature is the dynamic metabolic changes of tumor cells during distant metastasis, which enhance the nutrient availability of the metastatic niche through active adaptation or passive selection to modify their metabolic state. For instance, the uptake of lactate and pyruvate can facilitate the survival of circulating tumor cells by enhancing their resistance to oxidative stress (82). Researchers have discovered that the metabolic pattern within the same tumor tissue is affected by a variety of factors. Yuneva et al. found that different mutations in carcinogenic genes lead to differences in metabolic reprogramming in liver cancer, with the glucose uptake and catabolic metabolism of MYC-induced liver tumors significantly higher than those of MET-induced tumors (83). This phenomenon not only exists in glucose metabolism, but also in glutamine metabolism, where the level of glutamine in MET-induced tumors is nearly doubled, and further research has found that glutamine catabolic metabolism is increased in MYC-induced tumors, while glutamine synthesis is increased in MET-induced tumors (83). Among them, Krebs cycle glutamine metabolism is considered one of the foundations for the survival of over-expressed MYC cells. Research has shown that MYC can enhance the sensitivity of tumor cells to glutamine synthetase inhibitors, and researchers believe that MYC can serve as a biomarker for glutamine synthetase inhibitor therapy, the judgement of MYC expression level will provide more treatment opportunities. At the same time, the side effects caused by specific inhibition of glutamine synthetase are much smaller than those caused by glutamine analog therapy (84). The above research indicates that there is metabolic heterogeneity in different oncogenic gene-induced liver cancer, which will affect the generation and development of tumors, including ATP generation, ROS detoxification and other functions, and the corresponding phenotype will further affect the sensitivity of tumors to anti-cancer treatment. Concurrently, anti-cancer treatments can modulate the metabolic heterogeneity of hepatocellular carcinoma tissues. For instance, studies have demonstrated that sorafenib treatment leads to a significant enhancement in glucose uptake and lactate production, which is attributed to the overexpression of key glycolytic enzymes (85). Moreover, extensive research has shown that energy metabolism-related genes in hepatocellular carcinoma, such as NANOG, MYC, and CTNNB1, are also implicated in the development of sorafenib resistance (86–88).

Concurrently, on account of the variegated metabolic states exhibited by liver cancer at diverse stages, coupled with the low sensitivity of alpha-fetoprotein (AFP) in detecting early-stage liver cancer, researchers have carried out an analysis of the serum metabolomics of liver cancer patients during the early and late phases of the disease, discovering that the levels of alanine, glutamine, and lactate in the serum of patients with late-stage liver cancer are lower than those of patients with early-stage liver cancer. Alanine, glutamine, and lactate can contribute to identifying and optimizing the metabolic classification for HCC diagnosis (89). Meanwhile, the level of tyrosine is markedly lower in patients with advanced liver cancer than in normal persons, and researchers have signified that the level of tyrosine is an important serum biomarker for the progression of HCC (89). Researchers have analyzed liver cancer by leveraging transcriptomics data, genomic-scale metabolic data, and other methodologies, and have associated the subtypes with prognosis to construct predictive models (90). Particularly, in the realm of lipid metabolism, by adopting machine learning and integrating it with the tumor microenvironment, it is practicable to undertake risk assessment of liver cancer prognosis while conducting drug sensitivity studies, thereby facilitating patients in attaining personalized treatment. With the extensive employment of related new technologies such as liquid biopsy, Daniels et al. have discerned significant disparities in the metabolic abundance of uric acid and xylitol in HCC compared to normal tissue by analyzing saliva and plasma-related metabolic products. Such studies are expected to accomplish high-sensitivity non-invasive liver cancer screening in the future (91).

The liver cancer-related heterogeneity not only depends on the temporal dimension, but also on the spatial dimension. For example, Guo et al. identified three subtypes in liver cancer cells by integrating single-cell sequencing data and spatial transcriptomics data, namely, ARG1+ metabolism subtype (Metab-subtype), TOP2A+ proliferation phenotype (Prol-phenotype), and S100A6+ pro-metastatic subtype (EMT-subtype) (92). These subtypes exhibit different characteristics of metabolism, proliferation, and early metastasis. The different proportions of Metab-subtype and EMT-subtype will determine the tumor’s diverse proliferation and metastasis potential (92). In the future, we will be able to perform subtype-specific detection of tumors, which will help to more accurately predict the prognosis of liver cancer and determine relevant surgical indications.

## 3 The microenvironment of hepatocellular carcinoma

The tumor microenvironment (TME) plays a crucial role in various stages of cancer progression, including the initiation of cancer, epithelial-mesenchymal transition (EMT), invasion, and metastasis. TME provides nutrients for tumor cells, while its metabolic products also have an impact on TME. The main cellular components of the tumor microenvironment include hepatoma cells, hepatocytes, hepatic stellate cells (HSCs), myeloid-derived suppressor cells (MDSCs), cancer-associated fibroblasts (CAFs), tumor-associated macrophages (TAMs), tumor-associated neutrophils (TANs), immune cells (regulatory and cytotoxic T cells), endothelial cells, and various other cell types. Non-cellular components mainly consist of matrix produced by the above-mentioned relevant cells, including extracellular matrix (ECM) proteins, proteinases and their inhibitors, cytokines and growth factors. Interestingly, immune cells also exhibit complex metabolic patterns similar to those seen in tumors There is a significant difference in energy consumption between immune cells in resting and activated states (9). A large body of evidence suggests that metabolic reprogramming not only promotes tumor progression but also affects the microenvironment and its associated immune escape mechanisms. For instance, suppression of the demethylase FTO gene can attenuate the glycolytic activity of tumor cells, thereby reinstating the functionality of CD8^+^ T cells and impeding tumor growth (93).

Under normal physiological conditions, hepatic stellate cells (HSC) are usually in a quiescent state, primarily functioning to store retinyl esters or metabolites containing vitamin A (94). However, continuous chronic liver injury such as non-alcoholic fatty liver disease (NAFLD) and viral infection can lead to sustained activation of quiescent HSC, resulting in persistent hepatocellular damage. This is accompanied by increased extracellular matrix (ECM) synthesis and impaired fibrinolysis, leading to gradual disruption of the normal liver architecture and ultimately culminating in cirrhosis and hepatocellular carcinoma (95).

The liver, as an immune-exempt organ, contains a large number of macrophages, including resident Kupffer cells and recruited macrophages (96). Based on protein expression, secretion of cytokines and function, tumor-associated macrophages (TAMs) are usually divided into two subgroups: classically activated TAMs (M1-TAMs) and alternatively activated TAMs (M2 TAMs) (97). TAMs coordinate complex dynamic intercellular interactions with hepatocellular carcinoma (HCC) cells through various pathways such as cell-to-cell contact and soluble messengers (98). HCC cells recruit monocytes and promote their differentiation into M2-like phenotype TAMs by secreting various factors to maintain the immunosuppressive tumor microenvironment (99).

Under the influence of tumor-derived factors, myeloid cells are hijacked to become MDSCs, which not only suppress the anti-tumor function of T cells, but also accelerate tumor progression through promoting angiogenesis, cell invasion, and formation of metastatic pre-metastatic niches. The ability of MDSCs to inhibit the anti-tumor function of T cells and natural killer (NK) cells is closely associated with the clinical prognosis and treatment outcomes in patients with solid tumors (100).

In liver cancer tissues, Cancer-associated fibroblasts (CAFs) are the most abundant and critical component in the Tumor Microenvironment (TME). The extracellular matrix (ECM) produced by CAFs interacts with cancer cells to influence tumor growth and invasion (101). Researchers have discovered through single-cell sequencing and bioinformatics technology that the abundance of fibroblasts in HCC is higher than that in adjacent control tissues, while the abundance of endothelial cells is lower (102).

Therefore, targeting the crosstalk between tumors and relevant cells in the TME to improve the tumor immune microenvironment and enhance metabolic competition between tumors and the immune system will be a new focus for future research.

## 4 Metabolic interactions in the hepatocellular carcinoma tumor microenvironment

### 4.1 Metabolic crosstalk between hepatic stellate cells and hepatocellular carcinoma cells

Cancer cells maintain an alkaline intracellular environment by undergoing aerobic glycolysis, which generates a large amount of protons that are transported to the extracellular space through upregulation of H+ transport proteins. This, combined with the low perfusion of tumor tissue, leads to acidification of the local tumor microenvironment. Research has shown that metabolic reprogramming in tumors is involved in the activation and differentiation of hepatic stellate cells (HSCs), such as acidification of the microenvironment inducing HSC activation and differentiation into myofibroblasts in a P-Erk1/2-dependent manner (103). The significant activation of HSCs leads to overexpression of osteopontin (OPN) and contributes to hepatocellular carcinoma (HCC) metastasis. Meanwhile, the differentiated myofibroblasts infiltrate the HCC stroma and promote tumor progression (103–105).

Compared with quiescent HSC (qHSC), activated HSC (aHSC) exhibit higher rates of glucose utilization, transport capacity, and glycolytic activity (106). Proteomic analysis has demonstrated a significant increase in the expression of metabolic enzymes such as glucose transporter 1 (GLUT1), hexokinase 2 (HK2), and pyruvate kinase M2 (PKM2) in aHSC. Concurrently, there is sustained downregulation of gluconeogenic enzymes such as phosphoenolpyruvate carboxy kinase 1 (PCK1) and fructose-1,6-bisphosphatase 1 (FBP1) (107). The abundant lactate produced from glycolysis is divided into two pathways: intracellular accumulation inhibits proliferation and myofibroblast fibrogenesis-related gene expression, while inducing lipid accumulation and the expression of genes involved in lipogenesis (107). The other pathway involves upregulation of monocarboxylate transporter 4 (MCT4) to regulate extracellular lactate concentration, maintaining tumor microenvironment acidity and sustaining activation of HSC (108) In the process of activating HSC, gene expression regulating glutamine degradation is enhanced. Some researchers have found that by blocking glutamine degradation, they can inhibit mitochondrial respiration, cell growth, and migration of HSCs (109).

Rapid proliferation of liver cancer cells can deplete nutrients in the microenvironment, leading to sustained nutrient deprivation and decreased oxygen levels. In a continuous hypoxic environment, cancer cells are stimulated to secrete TGF-β and extracellular vesicles (EV) on a regular basis (110). The activation of HSC by TGF-β promotes epithelial-mesenchymal transition (EMT) in HCC to enhance its aggressiveness (111). The release of a large number of EV activates HSC and downregulates PTEN, thus enhancing the AKT and ERK signaling pathways to increase the proliferation and invasion capabilities of HSC (112). In a hypoxic environment, platelet-derived growth factor (PDGF) secretion by HSC is significantly increased to promote the proliferation of HCC cells, while it also alleviates bile acid (BA)-induced apoptosis in a PI3K/Akt signal transduction-dependent manner (113). Other studies have further demonstrated that PDGF brings about an increment in glycolytic metabolism within HSCs, which will exert an influence on the release of extracellular vesicles from HSCs and facilitate the progression of liver fibrosis (114). Additionally, hypoxia-inducible factor-1α (HIF-1α) enhances the secretion of miRNA from activated hepatic stellate cells under hypoxic and inflammatory conditions, promoting the invasion and metastasis of HCC cells and stimulating HSC activation (112, 115). Changes in nutrients in the microenvironment can affect relevant functions of HSCs, such as reducing the activation of human hepatic stellate cells by supplementing branched-chain amino acids and branched-chain α-keto acids (116).

In addition to the metabolic reprogramming of HCC and HSC mentioned above, which leads to a positive feedback loop in tumor metabolism, there is also lipid metabolism reprogramming during the activation and proliferation of HSC. This reprogramming provides the necessary lipids and prerequisites for membrane construction and post-translational modification of proteins. Studies have shown that exosomes derived from cancer cells not only regulate the activation of HSC through the PTEN/PDK1/Akt pathway, but also modulate the enzyme activity of lipid metabolism-related enzymes such as ATP citrate lyase (ACLY) and fatty acid synthase (FASN), indicating a positive correlation between exosomal content from cancer cells and lipid content in HSC (117).

In the tumor microenvironment, both HSC and HCC can secrete various cytokines and exosomes, mediate intercellular communication, and form positive feedback loops, collectively influencing the nature of the tumor microenvironment. For instance, HSCs facilitate the progression of HCC by regulating histone acetylation within the TME (118). This establishes a pro-metastatic environment suitable for tumor cell invasion and migration, promotes the formation of new vascular networks, and provides nutrients and oxygen for the tumor (119). Therefore, research on intercellular communication substances and their mechanisms of action will be the theoretical basis for future novel therapeutic targets in cancer treatment.

### 4.2 Metabolic crosstalk between hepatocellular carcinoma cells and cancer-associated fibroblasts

Currently, research on the function of cancer-associated fibroblasts (CAFs) in the tumor microenvironment (TME) has shown that they play a crucial role in the occurrence and development of hepatocellular carcinoma (HCC). CAFs interact with liver cancer to change the physical and chemical properties of TME, subsequently altering the biological behavior of both CAFs and tumor cells. The secretion of cytokines by CAFs has been implicated in poor prognosis and drug resistance in tumors (120, 121). Studies have indicated that cancer cells secrete hydrogen peroxide into the TME to induce oxidative stress in neighboring stromal cells (122). Consequently, cancer cells reprogram metabolism in CAFs, leading to the secretion of metabolites such as pyruvate and lactate which are then absorbed by cancer cells as a carbon source to meet their metabolic needs - a phenomenon known as “reverse Warburg effect” (123, 124). Shan et al. reported elevated expression levels of glycolysis-related enzymes such as lactate dehydrogenase A (LDHA), PKM2, and monocarboxylate transporter 4 (MCT4) in associated fibroblasts, further supporting the “reverse Warburg effect”. Despite this metabolic reprogramming, it was unexpected that proliferation efficiency for CAFs was slower compared to normal fibroblasts (NF), indicating that increased metabolic rates do not promote proliferation for CAFs (124). Zhang et al. found that decreased expression of isocitrate dehydrogenase 3 complex alpha subunit (IDH3α) is associated with metabolic reprogramming; upregulation of IDH3α expression can prevent NF from transforming into CAFs. Reduced IDH3α alters the ratio between α-KG and succinate/fumarate, stabilizing HIF-1α under normoxic conditions to promote glycolysis. Interestingly, cell adhesion to extracellular matrix also plays an important role in cellular metabolism: detachment from ECM inhibits glucose metabolism and mitochondrial respiration, leading to reduced energy synthesis within cells and increased intracellular ROS production (125).

The metabolic reprogramming behavior of cancer-associated fibroblasts (CAFs) is not limited to glucose metabolism, as CAFs and cancer-associated fibroblasts (PTFs) exhibit significant differences in lipid metabolism. Wang et al.’s study showed that the expression level of CD36 in CAFs is significantly higher than that in PTFs, and CD36 plays an important role in the biological regulation of CAFs, affecting tumor cell proliferation and migration by regulating lipid metabolism (126). Lipidomics and high-throughput mass spectrometry analysis revealed that the content of phosphatidic acid, phosphatidylglycerol, and phosphatidylserine is significantly higher in CAFs than in PTFs, while the content of phosphatidylcholine, lyso-phosphatidylcholine, and lyso-phosphatidylinositol is significantly lower in CAFs (126). These substances may affect the biological behavior of tumor cells. These substances may affect the biological behavior of tumor cells (127). Additionally, CAFs increase the unsaturated fatty acyl chains and membrane fluidity of lyso-phospholipids in tumor cells to enhance glucose uptake and metabolism (128). In hepatocellular carcinoma (HCC) cells, CD36 also promotes free fatty acid uptake and epithelial-mesenchymal transition (EMT), accelerating tumor progression and metastasis (129). Furthermore, the metabolic reprogramming of CAFs not only affects cancer cells but also impacts the immune environment within the tumor microenvironment (TME). For instance, CD36^+^CAF-mediated oxidized low-density lipoprotein (LDL) uptake induces macrophage migration inhibitory factor (MIF) expression dependent on MIF-CD74 signaling pathway which recruits bone marrow-derived suppressor cells(CD33^+^ MDSCS), leading to negatively regulating the immune system (130). Furthermore, HCC-related lipid products affect cellular status within CAFS. Mazzocca et al. stated that hepatoceullar carcinoma(HCC)-derived lysophosphatic acid(LPA) plays a critical role in promoting differentiation from PTFS into a CAF-like myofibrobast phenotype (131).

CAF and tumor cells both exhibit mutual interference in amino acid metabolism. CAFs demonstrate glutamine-dependent invasion, driving them to migrate from the low glutamine area at the core of the tumor to the high glutamine area. This suggests that low glutamine promotes CAF migration and invasion, which in turn facilitates tumor cell movement towards nutrient-rich areas (132). During glutamine-dependent migration, CAFs are regulated by TRAF6 and p62-mediated polarized distribution of AKT2. The polarization of AKT2 inhibits glutamine-driven CAF invasion and tumor cell escape (132). The crosstalk between CAFs and tumor cells in amino acid metabolism allows them to alleviate nutrient deficiency in TME while enabling efficient utilization of amino acids to promote tumor growth and development.

After being released by CAFs, extracellular vesicles are internalized by cancer cells and affect the tumor cell metabolism by transferring various substances. Metabolites associated with CAFs will be carried by their extracellular vesicles, including proteins and lipids, to enhance the nutrient metabolism and migration ability of tumor cells (133). For example, TUG1 in CAF-derived extracellular vesicles directly regulates the expression of glycolytic genes (such as HK2 and PKM2), glucose uptake, and lactate levels through the miR-524-5p/SIX1 axis (134). Studies have shown that CAFs upregulate levels of C-C motif chemokine ligands such as CCL2, CCL5, and CCL20 to coordinate activation of HCC cell ERK/PKM2 and TGF-β pathways to enhance tumor metastasis phenotype and regulate tumor metabolism (135, 136). Under normoxic conditions, for instance, CCL5 inhibits HIF-1α ubiquitination degradation while activating downstream factor ZEB1 to promote EMT and metastasis (137). Additionally, IL-6 derived from CAFs is a major source in the TME of different types of tumors. As a mediator of cancer cachexia syndrome, IL-6 activates the IL-6/JAK/STAT3 pathway to induce TG2 expression in order for tumors to acquire an EMT phenotype (124, 138).

In the TME, CAF mediates ECM formation by secreting enzymes associated with matrix remodeling. The function of ECM includes maintaining mechanical tension of the matrix and facilitating cell communication through cell membrane surface receptor integrins (124). Liu et al. demonstrated that increased ECM stiffness induces YAP activation, which on one hand promotes HCC cell migration and accelerates aerobic glycolysis (139). Furthermore, enhanced ECM stiffness induces amino acid exchange between cancer cells and their CAF, thereby promoting tumor proliferation (140). When tumor cells are detached from the ECM, glucose metabolism and mitochondrial respiration are inhibited, leading to reduced cellular energy synthesis and increased intracellular ROS levels, indicating the important role of ECM adhesion in cellular metabolism (125). These studies all indicate that CAF secretion influences ECM remodeling, while ECM remodeling simultaneously affects both CAF and HCC cells.

### 4.3 Metabolic crosstalk between hepatocellular carcinoma cells and tumor-associated macrophages

The metabolic products of tumor cell proliferation can act as signaling molecules to hijack the phenotype and function of infiltrating TAM, reprogramming their metabolism (141). In hepatocellular carcinoma (HCC), a large amount of lactate is produced in aerobic glycolysis and released into the tumor microenvironment (TME) through monocarboxylate transporter 4 (MCT4) (142). Lactate in TME is transported into macrophages via monocarboxylate transporter 1 (MCT1), where it generates pyruvate through lactate dehydrogenase (LDH), inhibiting the expression of prolyl hydroxylase (PH) and ultimately limiting the proteasomal ubiquitination degradation of HIF-1α (143). Hypoxia induces HIF-1α expression by increasing glucose metabolism through Toll-like receptor/NF-kB and PI3K/AKT/mTOR pathways, upregulating LDH and pyruvate dehydrogenase kinase to promote pyruvate metabolism for increased lactate concentration (141). Lactate, through HIF-1α, promotes M2 polarization of TAMs, upregulates vascular endothelial growth factor (VEGF) and arginase-1(ARG-1) expression; the former promotes angiogenesis while the latter catalyzes polyamine production to enhance cancer cell proliferation (144). The enhanced glycolysis in tumor cells and elevated lactate concentration in TME cooperate to enhance TAM immunosuppression and improve tumor perfusion (145).

Macrophages acquire exogenous lipids through phagocytosis, micropinocytosis, or recognition by scavenger receptors such as CD36 to obtain lipid droplets (146). The lysosomes then degrade the lipids into free cholesterol and fatty acids. The transfer of cholesterol from TAM membranes serves as a nutrient supply for cancer cells and enhances IL-4 signaling pathways while inhibiting interferon IFN-γ expression to promote tumorigenic effects (141, 146). After fatty acid oxidation, it participates in the TCA cycle. Studies have shown that M2-like macrophages are beneficial for fatty acid oxidation (FAO), while M1-like macrophages are involved in increasing fatty acid synthesis (FAS). With enhanced metabolism of HCC cells, competition for nutrients leads to increased FAO and decreased aerobic glycolysis, promoting ROS expression and inducing nuclear factor (Nrf2)-mediated M2 polarization (147, 148). Specific fatty acids and their metabolites have unique effects on TAM function, such as arachidonic acid and linoleic acid, both of which have been shown to promote a pro-tumor phenotype (146, 149). For example, arachidonic acid forms PGE2 through COX and PGE2 synthase, participating in regulating tumor proliferation, chemotherapy resistance, angiogenesis, immune suppression and other biological functions (150).

Hypoxia stimulates the release of a large amount of ATP into the TME, and high concentrations of ATP regulate Ca^2+^ signaling through P2Y11, which is specifically expressed in cancer cells, and enhance the migration ability of HCC cells (151, 152). Meanwhile, hypoxia will further give rise to M2 polarization of macrophages, particularly when tumor cells are co-cultured with macrophages (153). Meanwhile, ATP rapidly degrades to adenosine through pathways such as PI3K/Akt and MEK/ERK to regulate macrophage proliferation and induce adenosine accumulation to promote immune suppression (154). The polished result: Meanwhile, TAMs will infiltrate the hypoxic regions of the tumor preferentially, thereby enhancing the probability of immune evasion (155).

**Figure 2:**
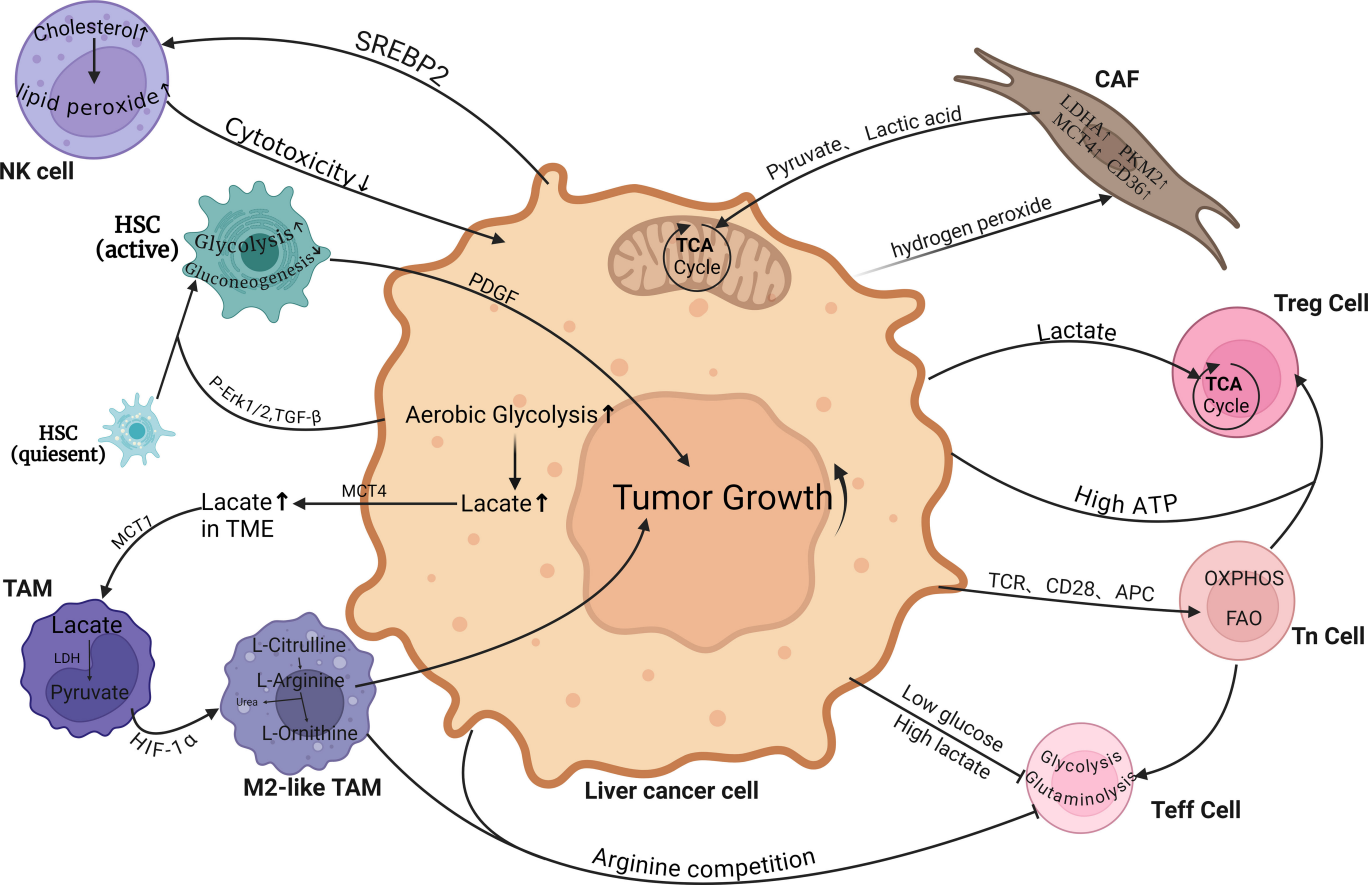
Metabolic crosstalk between hepatocellular carcinoma cells and various non-cancerous cells within the tumor microenvironment (TME). Tumor cells rely on the tumor microenvironment (TME) for nutrients and release metabolic byproducts that impact nearby immune and stromal cells, promoting immune exhaustion, inhibiting immune surveillance, and triggering epithelial-mesenchymal transition and angiogenesis. TAM, Tumor-associated macrophages; CAF, Cancer-associated fibroblasts; HSC, Hepatic Stellate Cells; Teff cell, Effector T cell; Tn cell, Naive T cell; Treg cell, Regulatory T cells. (Created with BioRender.com).

Unlike M1 TAM, M2 TAM does not necessitate glycolytic function as long as its OXPHOS machinery remains intact. This metabolic flexibility enables it to exhibit greater tolerance towards hypoxic environments and further induce the expression of M2-like markers (156). The M2 TAM marker is arginase 1 (Arg 1), which consumes arginine to form urea and ornithine. Ornithine is not only a precursor of polyamines and proline, but also derived from TAMs, which benefits tumor cell proliferation (157). Cancer cells secrete IL-10 to induce the B7-H1/PD-1 pathway on Kupffer cells, leading to impaired function of effector T cells (158) (**Figure 2**).

### 4.4 Metabolic crosstalk between hepatocellular carcinoma cells and tumor infiltrating lymphocytes

The metabolic reprogramming of T cells occurs during their self-activation process, transitioning rapidly from the characteristic metabolism of Tn cells, oxidative phosphorylation (OXPHOS), and fatty acid oxidation (FAO), to the characteristic metabolic processes of Teff cells, glycolysis and glutamine decomposition, in order to sustain growth and proliferation (159). Among them, amino acids exert a crucial role in the activity and anti-tumor capacity of T cells. For instance, glutamine facilitates the activation of the mammalian target of rapamycin complex 1 (mTORC1) target and the synthesis of glutathione, thereby supporting the functioning of T cell effectors (160). The aforementioned metabolic reprogramming is achieved through pathways such as TCR antigen stimulation, CD28 interaction with APC ligands, etc (161). Although Teff cells primarily rely on aerobic glycolysis, their mitochondrial tricarboxylic acid cycle (TCA) and OXPHOS continue to provide ATP and generate a certain level of reactive oxygen species (ROS) to maintain TCR signal transduction (162). Studies have shown that CD28 mediates the upregulation of relevant nutrient transporters’ expression and their translocation to the cell membrane; for example, an increase in monocarboxylate transporter (MCT) expression is observed in order to facilitate the clearance of lactate produced by glycolysis (163). In contrast, the main metabolic processes of Treg cells are ketone body mitochondrial oxidation and FAO oxidation; furthermore, the rate of glycolysis in Treg cells is much lower than that in Teff cells (164).

The TME environment with low glucose and high lactate will inhibit the proliferation and function of Teff cells. For example, IFN-α establishes a high-glucose environment by inhibiting HCC cell glycolysis to trigger mTOR signaling activation, enhancing the cytotoxicity of T cells (165). Compared to Teff cells, Treg cells transport lactate through MCT1 to supply their own TCA cycle and also produce phosphoenolpyruvate (PEP) (166). Tregs demonstrate metabolic flexibility, which enables metabolism to shift towards OXPHOS, allowing them to tolerate high lactate levels within the TME and effectively acquire nutrients from the TME to support tumor proliferation promotion and immune tolerance (167). The above-mentioned lactate is involved in Treg cell metabolism and promotes the nuclear translocation of nuclear factor of activated T-cells NFAT1 and enhances PD-1 expression, which suggests poor response to immunotherapy (168). Upon activation of NK cells, glucose turns into the principal metabolic fuel, and an increasing amount of evidence implies that NK cell toxicity is positively correlated with glycolysis (169). Nevertheless, the competitive inhibition of glucose metabolism by tumors results in a lower maintenance level of glycolysis in NK cells, leading to a reduction in cytotoxicity and cytokine production and thereby facilitating immune evasion (170). *In vitro* experiments have shown that lactate reduces perforin and granzyme expression in NK cells, leading to decreased cytolytic function and cytotoxic activity (171). Energy substance depletion is not limited to glucose; cancer cells compete for methionine through SLC43A2, which impairs STAT5 expression in T cells, affecting histone methylation and their function. Both tumor cells and M2-like TAMs consume exogenous arginine in the TME extensively, inhibiting the proliferation and function of T cells (172, 173). High ATP concentration in the TME enhances the chemotaxis of Treg cells while selectively increasing immunosuppressive cells (174). In lipid metabolism processes, hepatocytes drive excessive cholesterol production through sterol regulatory element-binding protein 2 (SREBP2), inducing lipid peroxide accumulation and inadequate cytotoxicity (175). Meanwhile, metabolic reprogramming is bound to affect the distribution of T cell subpopulations within the TME, where diverse types of T cells play disparate roles in the advancement of liver cancer. In contrast to CD8^+^T cells, CD4^+^T cells play a distinct role in the progression of liver cancer (176). It has been reported that linoleic acid selectively induces the loss of liver CD4^+^T lymphocytes, thereby undermining the anti-tumor surveillance function. Meanwhile, linoleic acid can enhance the expression of carnitine palmitoyltransferase (CPT), thereby accelerating the development of HCC (177, 178). However, some studies suggest that the issue of T cell function impairment caused by linoleic acid deficiency resulting from the competition between liver cancer and T cells for polyunsaturated fatty acids, especially linoleic acid (179). Therefore, it is imperative to further elucidate the role of immune cells related to liver cancer in the TME, such as whether T cells have a nutrient-dependent or other related influencing factors.

In conclusion, tumors compete with T cells for nutrients on one hand and influence the function and proliferation of T cells through their own metabolic by-products on the other hand. Therefore, studying the mutual interference between cancer cell metabolism reprogramming and CTLs, as well as clarifying the roles of relevant nutrients and their metabolites in the anti-tumor immune process, may improve the therapeutic effects of existing treatment methods and reveal new potential therapeutic targets for further research.

### 4.5 Metabolic crosstalk between hepatocellular carcinoma cells and myeloid-derived suppressor cells

MDSCs are rapidly expanding in cancer, consisting of two main subgroups: monocytic MDSCs (M-MDSC) and granulocytic MDSCs (G-MDSC), which share the ability to maintain an immature state and suppress adaptive immunity. MDSCs derived from tumors exhibit increased rates of glycolysis and upregulation of glycolytic genes. The metabolic byproduct phosphoenolpyruvate (PEP) can act as an antioxidant to prevent ROS-mediated cell apoptosis (180). Studies have shown that the quantity of immunosuppressive MDSCs increases when cultured in high concentrations of extracellular lactate salt (171). Recent research on metabolic reprogramming in MDSCs has revealed that they utilize fatty acid oxidation (FAO) as their primary energy source, characterized by increased mitochondrial mass and fatty acid uptake (181). This reprogramming is driven by the induction of the STAT3 and STAT5 pathways, which are triggered by tumor-derived cytokines such as G-CSF and GM-CSF (167). Furthermore, there is a positive correlation between upregulated FAO and enhanced immunosuppression, indicating that FAO not only serves as a cellular energy source for MDSCs but also acts as the primary metabolic fuel for producing inhibitory cytokines (100).

In the metabolism of amino acids, G-MDSCs upregulate the expression of cationic amino acid transporter 2 (CAT2) and arginase to increase the uptake of L-Arg. L-Arg starvation will lead to antigen recognition failure in T cells and loss of anti-tumor function (182, 183). M-MDSCs express high levels of iNOS, which, in synergy with L-Arg starvation, arrest T cells in the G0-G1 phase of the cell cycle and directly promote apoptosis in T cells (184). In light of this characteristic, researchers have employed All-trans-retinoic acid (ATRA) to markedly reduce the production of Arg1 and iNOS, and reverse the accumulation of MDSCs within the tumor (185). In terms of cysteine (L-Cys) metabolism, one pathway utilized by MDSCs involves upregulating solute carrier family member 11 (SLC7A11) to chelate cysteine and convert it into L-Cys. Another pathway involves catalyzing methionine synthesis into L-Cys, but neither pathway results in the export of L-Cys (186). Some portion of cysteine released by APC is metabolized by MDSCs, which limits extracellular cysteine concentration and indirectly inhibits T cell activation.

Extracellular adenosine (eADO) is generated by the ATP dephosphorylation mediated by highly expressed ectonucleoside triphosphate diphosphohydrolase 1 (CD39) and ecto-5’-nucleotidase (CD73) in MDSCs. It accumulates at high concentrations under hypoxic conditions, inducing A2AR to impair the function and proliferation of T and NK cells, while also stimulating the expression of Foxp3 in Treg cells, leading to immune evasion in cancer and resulting in adverse clinical outcomes (187). Previous studies have found that IL-37 significantly promotes glycolysis and OXPHOS levels in MDSCs within the HCC tumor microenvironment to increase ATP production and release, with ATP binding to P2X7R inhibiting the immunosuppressive function of MDSCs (188). However, these findings appear contradictory, and further research into the molecular mechanisms and signaling pathways involved is needed to potentially turn off the immunosuppressive function of MDSCs, making it a new therapeutic target for improving patient prognosis.

## 5 The clinical application and prospects of metabolic therapy for liver cancer

Currently, with the in-depth exploration by numerous researchers into the specific mechanisms of liver cancer metabolic reprogramming, the use of targeted inhibitors for certain specific metabolic steps to achieve targeted therapy for liver cancer has emerged as a new hotspot in the field of liver cancer treatment research. Currently, the discovery of classic drugs targeting the metabolic processes of liver cancer is of significant importance for the treatment of liver cancer. For instance, the diabetes drug metformin, which was mentioned previously, has several clinical trials ongoing (NCT03184493, NCT04033107, NCT04114136). Meanwhile, a multicenter cohort study targeting patients with unresectable HCC demonstrated that the combined treatment of metformin and sorafenib significantly enhanced the long-term survival rate (adjusted hazard ratio = 3.464; P < 0.001) (189).

Apart from conventional drugs, a considerable number of specific inhibitors are in the course of continuous development. The first promising therapeutic target within the glycolytic process is the first rate-limiting enzyme, namely HK2. Lonidamine was initially tested on animals in 1996; however, its severe liver and pancreatic toxicity restricted its clinical application (190). Another HK2 inhibitor, 2-deoxyglucose (2-DG), functioning as a glucose analog, operates through non-competitive inhibition of HK2 activity (19). In preclinical studies, it was discovered that 2-DG in combination with sorafenib could synergistically enhance anti-cancer efficacy (191, 192). In addition to the aforementioned HK2 inhibitors, natural extracts and their analogues such as Chrysin and Methyl Jasmonate are also in the preclinical research stage, although they might not have entered clinical trials due to side effects and other influencing factors (193, 194). PKM2 is the rate-limiting enzyme at the end of glycolysis, and TEPP-46, as an activator of pyruvate kinase, guides the sugar metabolism towards the tricarboxylic acid cycle to reduce lactate production, significantly diminishing HSC activation and liver fibrosis (195). Regarding LDHA, Ye et al. investigated a novel Pickering emulsion gel (APEG) loaded with oxaliplatin (OXA) and the LDHA inhibitor GSK2837808A (GSK) to confirm that the combination formulation can effectively enhance drug delivery efficiency and boost anti-tumor therapy by activating the tumor immune microenvironment and increasing the infiltration of CD8^+^T cells (196). Currently, inhibitors are not restricted to a single metabolic target. Emodin, a compound derived from Polygonum multiflorum Thunb, has been discovered to inhibit multiple metabolism-related enzymes, such as PKM2, HK2, and LDHA (197). Some researchers have found that Selective serotonin reuptake inhibitors (SSRIs) have an antidepressant, with their specific targeting of GLUT1 resulting in a reduction in glucose metabolism and a synergistic effect with anti-PD-1 therapy (198). In addition to directly targeting enzymes related to glucose metabolism, it is also feasible to target common factors influencing glucose metabolism, such as HIF-1α. HIF-1α promotes the process of glucose metabolism and activates the expression of multiple enzymes related to glucose metabolism. An anti-HIF-1α agent, EZN-2968, a reverse complementary oligonucleotide inhibitor, has been demonstrated to block the interaction between multiple myeloma plasma cells and their microenvironment and is currently in late-stage clinical trials (NCT01120288) to verify its safety and efficacy. Similarly, the HIF-1α mRNA inhibitor RO7070179 has also entered clinical trials and is currently in phase Ib for mechanism validation (NCT02564614). To restrict the function of Treg cells and the lactic acid level in the TME, the monocarboxylate transporter 1 (MCT1) inhibitor AZD3965 has entered clinical trials (NCT01791595) to verify the maximum safe dose and potential side effects (199).

In the lipid metabolism process, the analog of the SLC25A1 inhibitor PTL, namely Dimethylamino parthenolide (DMAPT), as a novel anti-cancer drug, has undergone relatively extensive research in pancreatic cancer and breast cancer (200, 201). Unlike SLC25A1 inhibitors that remain in the preclinical research stage, the ACLY inhibitor bempedoic acid has been approved by the FDA as a new lipid-lowering drug. Studies have indicated that bempedoic acid can inhibit liver steatosis and reduce the invasion and proliferation of cancer cells in breast cancer and pancreatic cancer (202, 203). Given that ACLY inhibitors are frequently associated with lipid regulation, there are currently several small molecule ACLY inhibitors in the process of clinical trials. Whether they will exert an effect on liver cancer patients or patients with precancerous lesions in the future still requires exploration and testing. Acetyl-coa carboxylase 1 (ACC1) is A key enzyme in fatty acid synthesis, which carboxylates with acetyl-coa carboxylate to form malonyl-coa. Its role in fatty acid synthesis makes it A promising therapeutic target in various metabolic diseases such as non-alcoholic fatty liver disease, and A potential choice for anti-tumor therapy (204). The anti-NASH effects of ACC1 inhibitors used alone or in combination with ACC1 inhibitors (PF-05221304)and glycerol-3-phosphate dehydrogenase2 (DGAT2) inhibitors (PF-06865571) have been tested in clinical trials (NCT03248882, NCT03776175). The results demonstrate that the use of ACC1 inhibitors alone has a significant inhibitory effect on NASH; however, its side effects are prone to cause hyperlipidemia. Meanwhile, the combination of ACC1 inhibitors with DGAT2 inhibitors can alleviate this side effect (205). Besides the aforementioned ACLY inhibitors, there are numerous pathways involved in fatty acid metabolism, and there are abundant metabolic targets. Some researchers believe that HMGCR inhibitors such as statins also influence the occurrence and development of liver cancer, and the clinical trial of pravastatin has entered the stage (NCT01075555).

Regarding glutamine metabolism, CB-839, a selective GLS1 inhibitor, has not demonstrated significant side effects in preclinical studies and is currently undergoing multiple clinical trials in lung cancer (NCT04250545) and pancreatic ductal adenocarcinoma (NCT03965845). Not merely restricted to the use of CB-839 alone, Jin et al. discovered that the combination of CB-839 and V-9302, a novel ASCT2 inhibitor, induces ROS and leads to cell apoptosis, and has also exhibited tumor inhibitory effects in a mouse model (206). In addition to the direct inhibition of metabolic enzymes, amino acid depletion therapy can also be employed to suppress related metabolic enzymes. Given the strong reliance of most tumor cells on exogenous arginine, investigators utilized pegylated recombinant human arginase (PEG-BCT-100) to deplete exogenous arginine (NCT01092091). Studies have indicated that PEG-BCT-100 is well tolerated in HCC, and the clinical benefits of ASS-negative patients are significantly superior to those of ASS-positive patients. The researchers also suggested that ASS negativity is a potential prognostic biomarker for HCC (207).

Presently, research on inhibitors of tumor metabolic processes is highly prevalent; however, due to factors such as drug toxicity and clinical efficacy, it proves challenging for related inhibitors to enter the large-scale clinical trial phase. Nevertheless, studies have indicated that combined treatment involving related inhibitors and drugs like sorafenib can effectively enhance patient outcomes. Hence, it is anticipated that in the future, with the advancement of drug delivery technology and the reduction of drug toxicity, these inhibitors can be applied in clinical practice to improve the prognosis of liver cancer patients and mitigate their drug resistance.

## 6 Outlook

Tumor cells augment their self-sustaining capacity by modulating nutrient uptake and energy metabolism to fulfill their own proliferation requirements. In the context of tumor immune activation, the participation of energy and metabolic intermediates, along with a multitude of biosynthetic processes, is indispensable. The evasion of immune surveillance by tumor cells involves the establishment of an immunosuppressive microenvironment through the secretion of metabolic byproducts and associated cytokines. During the process of metabolic reprogramming in liver cancer, a substantial quantity of nutrients and metabolites, including lactate, glutamine, and arginine, are released or transported. These molecules collectively reshape the tumor microenvironment (TME), thereby facilitating the progression of liver cancer. During the process of reshaping the tumor microenvironment (TME), active or passive reprogramming of tumor cells exerts an influence on the metabolism and self-renewal differentiation of non-hepatic cancer cells. In the aforementioned process, multiple signaling pathways play a pivotal role, with each key signaling molecule potentially serving as a novel target or biomarker for liver cancer treatment and early diagnosis, thereby offering valuable assistance. For instance, inhibition of MCT4 can impede lactate accumulation in the tumor microenvironment (TME) and augment CD8^+^ T cell-mediated anti-tumor immune response in hepatocellular carcinoma (HCC) (208). During tumor microenvironment (TME) remodeling, exosomes play a crucial role in intercellular communication and material exchange. Researchers have harnessed exosomes as carriers for targeted delivery technology. For instance, Wang et al. utilized exosomes as gene delivery vectors to successfully deliver short interfering RNA (siRNA) to target cancer cells, thereby inhibiting tumor metabolism and reversing oxaliplatin resistance (209). However, the therapeutic application of exosomes still faces numerous challenges. Existing studies have demonstrated that the source of exosomes significantly influences their properties. For example, tumor-derived exosomes (TDEs) express multiple receptors and ligands on their surface, enabling them to target actively growing *in situ* tumors and induce robust cytotoxic T cell responses (210). In contrast, exosomes from bone marrow-derived stem cells exhibit pro-tumor characteristics (211). If the differences among exosomes from various sources can be addressed in the future, this will facilitate personalized targeted therapy for different cell subtypes within tumor tissues.

However, it has been observed through research that in the event of a blockade or elimination of a specific metabolic pathway, tumor cells exhibit the ability to employ alternative pathways or energy sources. This dynamic metabolic alteration represents a survival mechanism employed by cancer cells, as the metabolic milieu within the TME gradually evolves alongside tumor progression. The targeted metabolic therapy for diverse pathways at different stages of tumor development holds immense significance; however, its research encounters substantial challenges. Currently, research has demonstrated that the spatial distribution of cells within the tumor microenvironment (TME) plays a significant role in shaping the immunodynamics of intricate tumors (212). This article examines metabolic crosstalk as a pivotal determinant influencing cell spatial distribution and proposes that targeting relevant metabolic targets in liver cancer treatment could potentially modulate cell distribution within TME, thereby enhancing prognosis. However, drugs targeting the corresponding metabolic pathways not only impact tumor cells but also exert effects on healthy proliferating cells, potentially leading to significant adverse reactions. The existing research has demonstrated that the utilization of nanoparticles, such as polymers and lipids, holds promise for enhancing drug solubility and achieving targeted delivery to minimize adverse effects (213). Despite the numerous challenges associated with nanoparticles, their distinctive capacity to target multiple sites, regulate the microenvironment, and facilitate safer drug delivery will continue to be the primary choice for HCC treatment. Therefore, future studies on liver cancer metabolism should focus on three main directions: firstly, a comprehensive analysis of the reprogramming of liver cancer metabolism and related metabolic networks in the tumor microenvironment (TME); secondly, investigation of the interactions between liver cancer metabolism-related targets and drug resistance; finally, development of targeted drugs against relevant metabolic pathways to enhance tumor immunity and facilitate clinical translation. In relation to the aforementioned research direction, this article posits that the field of liver cancer metabolic crosstalk holds promise for delivering personalized and less toxic treatment modalities to patients in the foreseeable future. Simultaneously, its research findings may unveil novel early diagnostic indicators with heightened specificity and staging significance.

## References

[1] Anderson NM Simon MC. The tumor microenvironment. Curr Biol. (2020) 30:R921–5. doi: 10.1016/j.cub.2020.06.081 PMC8194051 32810447

[2] Wei CY Zhu MX Zhang PF Huang XY Wang JK Yang XZ. PKCα/ZFP64/CSF1 axis resets the tumor microenvironment and fuels anti-PD1 resistance in hepatocellular carcinoma. J Hepatol. (2022) 77(1):163–76. doi: 10.1016/j.jhep.2022.02.019 35219791

[3] Liu Y Xun Z Ma K Liang S Li X Zhou S. Identification of a tumour immune barrier in the HCC microenvironment that determines the efficacy of immunotherapy. J Hepatol. (2023) 78:770–82. doi: 10.1016/j.jhep.2023.01.011 36708811

[4] Sung H Ferlay J Siegel RL Laversanne M Soerjomataram I Jemal A. Global cancer statistics 2020: GLOBOCAN estimates of incidence and mortality worldwide for 36 cancers in 185 countries. CA Cancer J Clin. (2021) 71:209–49. doi: 10.3322/caac.21660 33538338

[5] Llovet JM Kelley RK Villanueva A Singal AG Pikarsky E Roayaie S. Hepatocellular carcinoma. Nat Rev Dis Primers. (2021) 7:6. doi: 10.1038/s41572-020-00240-3 33479224 PMC13537433

[6] Wang Y Xia Y Lu Z. Metabolic features of cancer cells. Cancer Commun. (2018) 38:1–6. doi: 10.1186/s40880-018-0335-7 PMC6235388 30376896

[7] Fang Y Zhan Y Xie Y Du S Chen Y Zeng Z. Integration of glucose and cardiolipin anabolism confers radiation resistance of HCC. Hepatology. (2022) 75(6):1386–401. doi: 10.1002/hep.32177 PMC9299851 34580888

[8] Wilde L Roche M Domingo-Vidal M Tanson K Philp N Curry J. Metabolic coupling and the Reverse Warburg Effect in cancer: Implications for novel biomarker and anticancer agent development. Semin Oncol. (2017) 44:198–203. doi: 10.1053/j.seminoncol.2017.10.004 29248131 PMC5737780

[9] Sun H Li X. Metabolic reprogramming in resting and activated immune cells. Metabolomics (Los Angel). (2017) 7:188. doi: 10.4172/2153-0769.1000188 32655978 PMC7351252

[10] Wu L Zhang X Zheng L Zhao H Yan G Zhang Q. RIPK3 orchestrates fatty acid metabolism in tumor-associated macrophages and hepatocarcinogenesis. Cancer Immunol Res. (2020) 8:710–21. doi: 10.1158/2326-6066.CIR-19-0261 32122992

[11] Anderson NM Mucka P Kern JG Feng H. The emerging role and targetability of the TCA cycle in cancer metabolism. Protein Cell. (2018) 9:216–37. doi: 10.1007/s13238-017-0451-1 PMC5818369 28748451

[12] Stacpoole PW. Therapeutic targeting of the pyruvate dehydrogenase complex/pyruvate dehydrogenase kinase (PDC/PDK) axis in cancer. JNCI: J Natl Cancer Inst. (2017) 109(11). doi: 10.1093/jnci/djx071 29059435

[13] Ma X Li C Sun L Huang D Li T He X. Lin28/let-7 axis regulates aerobic glycolysis and cancer progression via PDK1. Nat Commun. (2014) 5:5212. doi: 10.1038/ncomms6212 25301052

[14] DeWaal D Nogueira V Terry AR Patra KC Jeon S-M Guzman G. Hexokinase-2 depletion inhibits glycolysis and induces oxidative phosphorylation in hepatocellular carcinoma and sensitizes to metformin. Nat Commun. (2018) 9:446. doi: 10.1038/s41467-017-02733-4 29386513 PMC5792493

[15] Upadhyay M Samal J Kandpal M Singh OV Vivekanandan P. The Warburg effect: Insights from the past decade. Pharmacol Ther. (2013) 137:318–30. doi: 10.1016/j.pharmthera.2012.11.003 23159371

[16] Sun H-W Yu X-J Wu W-C Chen J Shi M Zheng L. GLUT1 and ASCT2 as predictors for prognosis of hepatocellular carcinoma. PloS One. (2016) 11(12):e0168907. doi: 10.1371/journal.pone.0168907 28036362 PMC5201247

[17] Kim YH Jeong DC Pak K Han M-E Kim J-Y Liangwen L. SLC2A2 (GLUT2) as a novel prognostic factor for hepatocellular carcinoma. Oncotarget. (2017) 8:68381–92. doi: 10.18632/oncotarget.20266 PMC5620264 28978124

[18] Amann T Maegdefrau U Hartmann A Agaimy A Marienhagen J Weiss TS. GLUT1 expression is increased in hepatocellular carcinoma and promotes tumorigenesis. Am J Pathol. (2009) 174(4):1544–52. doi: 10.2353/ajpath.2009.080596 PMC2671384 19286567

[19] Du D Liu C Qin M Zhang X Xi T Yuan S. Metabolic dysregulation and emerging therapeutical targets for hepatocellular carcinoma. Acta Pharm Sin B. (2022) 12:558–80. doi: 10.1016/j.apsb.2021.09.019 PMC8897153 35256934

[20] Kaji K Nishimura N Seki K Sato S Saikawa S Nakanishi K. Sodium glucose cotransporter 2 inhibitor canagliflozin attenuates liver cancer cell growth and angiogenic activity by inhibiting glucose uptake. Int J Cancer. (2018) 142:1712–22. doi: 10.1002/ijc.31193 29205334

[21] Cham CM Gajewski TF. Glucose availability regulates IFN-γ Production and p70S6 kinase activation in CD8+ Effector T cells. J Immunol. (2005) 174:4670–7. doi: 10.4049/jimmunol.174.8.4670 15814691

[22] Lei Y Han P Chen Y Wang H Wang S Wang M. Protein arginine methyltransferase 3 promotes glycolysis and hepatocellular carcinoma growth by enhancing arginine methylation of lactate dehydrogenase A. Clin Trans Med. (2022) 12:e686. doi: 10.1002/ctm2.686 PMC8797063 35090076

[23] Zhao Y Li M Yao X Fei Y Lin Z Li Z. HCAR1/MCT1 regulates tumor ferroptosis through the lactate-mediated AMPK-SCD1 activity and its therapeutic implications. Cell Rep. (2020) 33:108487. doi: 10.1016/j.celrep.2020.108487 33296645

[24] Amelio I Cutruzzolá F Antonov A Agostini M Melino G. Serine and glycine metabolism in cancer. Trends Biochem Sci. (2014) 39:191–8. doi: 10.1016/j.tibs.2014.02.004 PMC3989988 24657017

[25] Li Z Zhang H. Reprogramming of glucose, fatty acid and amino acid metabolism for cancer progression. Cell Mol Life Sci. (2016) 73:377–92. doi: 10.1007/s00018-015-2070-4 PMC11108301 26499846

[26] Liu S Sun Y Jiang M Li Y Tian Y Xue W. Glyceraldehyde-3-phosphate dehydrogenase promotes liver tumorigenesis by modulating phosphoglycerate dehydrogenase. Hepatology. (2017) 66:1234–45. doi: 10.1002/hep.29202 28387968

[27] Wang H Lin F Xu Z Yu S Li G Liao S. ZEB1 transcriptionally activates PHGDH to facilitate carcinogenesis and progression of HCC. J Clin Gastroenterol Hepatol. (2023) 16:248–62. doi: 10.1016/j.jcmgh.2023.06.006 PMC10469392 37331567

[28] Adeva-Andany MM Pérez-Felpete N Fernández-Fernández C Donapetry-García C Pazos-García C. Liver glucose metabolism in humans. Biosci Rep. (2016) 36:e00416. doi: 10.1042/BSR20160385 27707936 PMC5293555

[29] Nogueira V Hay N. Molecular pathways: reactive oxygen species homeostasis in cancer cells and implications for cancer therapy. Clin Cancer Res. (2013) 19:4309–14. doi: 10.1158/1078-0432.CCR-12-1424 PMC3933310 23719265

[30] Yin X Tang Z Zhang L. ID1 promotes hepatocellular carcinoma proliferation and confers chemoresistance to oxaliplatin by activating pentose phosphate pathway. Clin Cancer Res. (2017) 36:166. doi: 10.1186/s13046-017-0637-7 PMC5701377 29169374

[31] Liu Y. The role of ASCT2 in cancer: a review. Eur J Pharmacol. (2018) 834:1–7. doi: 10.1016/j.ejphar.2018.07.007 30025811

[32] Yang L Venneti S Nagrath D. Glutaminolysis: A hallmark of cancer metabolism. Annu Rev BioMed Eng. (2017) 19:163–94. doi: 10.1146/annurev-bioeng-071516-044546 28301735

[33] Yu D Shi X Meng G Chen J Yan C Jiang Y. Kidney-type glutaminase (GLS1) is a biomarker for pathologic diagnosis and prognosis of hepatocellular carcinoma. Oncotarget. (2015) 6:7619–31. doi: 10.18632/oncotarget.3196 PMC4480704 25844758

[34] Zeng Y Jiang H Zhang X Xu J Wu X Xu Q. Canagliflozin reduces chemoresistance in hepatocellular carcinoma through PKM2-c-Myc complex-mediated glutamine starvation. Free Radic Biol Med. (2023) 208:571–86. doi: 10.1016/j.freeradbiomed.2023.09.006 37696420

[35] López de la Oliva AR Campos-Sandoval JA Gómez-García MC Cardona C Martín-Rufián M Sialana FJ. Nuclear translocation of glutaminase GLS2 in human cancer cells associates with proliferation arrest and differentiation. Sci Rep. (2020) 10(1):58264. doi: 10.1038/s41598-020-58264-4 PMC7010782 32042057

[36] Suzuki S Venkatesh D Kanda H Nakayama A Hosokawa H Lee E. GLS2 is a tumor suppressor and a regulator of ferroptosis in hepatocellular carcinoma. Cancer Res. (2022) 82(18):3209–222. doi: 10.1158/0008-5472.CAN-21-3914 PMC11057045 35895807

[37] Dias MM Adamoski D Dos Reis LM Ascenção C de Oliveira KR Mafra AC. GLS2 is protumorigenic in breast cancers. Oncogene. (2020) 39(3):690–702. doi: 10.1038/s41388-019-1007-z 31541193

[38] Matsuno T Goto I. Glutaminase and glutamine synthetase activities in human cirrhotic liver and hepatocellular carcinoma. Cancer Res. (1992) 52(5):1192–4. doi: 10.1158/0008-5472 1346587

[39] Ye Y Yu B Wang H Yi F. Glutamine metabolic reprogramming in hepatocellular carcinoma. Front Mol Biosci. (2023) 10:1242059. doi: 10.3389/fmolb.2023.1242059 37635935 PMC10452011

[40] Marsico M Santarsiero A Pappalardo I Convertini P Chiummiento L Sardone A. Mitochondria-mediated apoptosis of HCC cells triggered by knockdown of glutamate dehydrogenase 1: perspective for its inhibition through quercetin and permethylated anigopreissin A. Biomedicines. (2021) 9:1664. doi: 10.3390/biomedicines9111664 34829892 PMC8615521

[41] You H-J Li Q Ma L-H Wang X Zhang H-Y Wang Y-X. Inhibition of GLUD1 mediated by LASP1 and SYVN1 contributes to hepatitis B virus X protein-induced hepatocarcinogenesis. J Mol Cell Biol. (2024) 16(2):123–35. doi: 10.1093/jmcb/mjae014 PMC11440430 38587834

[42] Cruzat V Macedo Rogero M Noel Keane K Curi R Newsholme P. Glutamine: metabolism and immune function, supplementation and clinical translation. Nutrients. (2018) 10:1564. doi: 10.3390/nu10111564 30360490 PMC6266414

[43] Zhou Y Yu H Cheng S Chen Y He L Ren J. Glutamate dehydrogenase 1 mediated glutaminolysis sustains HCC cells survival under glucose deprivation. J Cancer. (2022) 13:1061–72. doi: 10.7150/jca.64195 PMC8824882 35154470

[44] Schiliro C Firestein BL. Mechanisms of metabolic reprogramming in cancer cells supporting enhanced growth and proliferation. Cells. (2021) 10:1056. doi: 10.3390/cells10051056 33946927 PMC8146072

[45] Dang CV Le A Gao P. MYC-induced cancer cell energy metabolism and therapeutic opportunities. Clin Cancer Res. (2009) 15:6479–83. doi: 10.1158/1078-0432.CCR-09-0889 PMC2783410 19861459

[46] Villar VH Merhi F Djavaheri-Mergny M Durán RV. Glutaminolysis and autophagy in cancer. Autophagy. (2015) 11:1198–208. doi: 10.1080/15548627.2015.1053680 PMC4590661 26054373

[47] Ying L Cheng M Lu Y Tao Q Chen X Shen B. Glutamine metabolism scoring predicts prognosis and therapeutic resistance in hepatocellular carcinoma. Oncol Res. (2021) 27:1610075. doi: 10.3389/pore.2021.1610075 PMC8724684 34992505

[48] Chiu M Tardito S Pillozzi S Arcangeli A Armento A Uggeri J. Glutamine depletion by crisantaspase hinders the growth of human hepatocellular carcinoma xenografts. Br J Cancer. (2014) 111:1159–67. doi: 10.1038/bjc.2014.425 PMC4453854 25072259

[49] Menendez JA Lupu R. Fatty acid synthase and the lipogenic phenotype in cancer pathogenesis. Nat Rev Cancer. (2007) 7:763–77. doi: 10.1038/nrc2222 17882277

[50] Baenke F Peck B Miess H Schulze A. Hooked on fat: the role of lipid synthesis in cancer metabolism and tumour development. Dis Model Mech. (2013) 6:1353–63. doi: 10.1242/dmm.011338 PMC3820259 24203995

[51] Koundouros N Poulogiannis G. Reprogramming of fatty acid metabolism in cancer. Br J Cancer. (2020) 122:4–22. doi: 10.1038/s41416-019-0650-z 31819192 PMC6964678

[52] Pope ED Kimbrough EO Vemireddy LP Surapaneni PK Copland JA Mody K. Aberrant lipid metabolism as a therapeutic target in liver cancer. Expert Opin Ther Targets. (2019) 23:473–83. doi: 10.1080/14728222.2019.1615883 PMC6594827 31076001

[53] Liu H-H Xu Y Li C-J Hsu S-J Lin X-H Zhang R. An SCD1-dependent mechanoresponsive pathway promotes HCC invasion and metastasis through lipid metabolic reprogramming. Mol Ther. (2022) 30:2554–67. doi: 10.1016/j.ymthe.2022.03.015 PMC9263248 35358687

[54] Hall Z Chiarugi D Charidemou E Leslie J Scott E Pellegrinet L. Lipid remodeling in hepatocyte proliferation and hepatocellular carcinoma. Hepatology. (2021) 73:1028–44. doi: 10.1002/hep.31391 32460431

[55] Röhrig F Schulze A. The multifaceted roles of fatty acid synthesis in cancer. Nat Rev Cancer. (2016) 16:732–49. doi: 10.1038/nrc.2016.89 27658529

[56] Rysman E Brusselmans K Scheys K Timmermans L Derua R Munck S. *De novo* lipogenesis protects cancer cells from free radicals and chemotherapeutics by promoting membrane lipid saturation. Cancer Res. (2010) 70:8117–26. doi: 10.1158/0008-5472.CAN-09-3871 20876798

[57] Zhang Z Qiao Y Sun Q Peng L Sun L. A novel SLC25A1 inhibitor, parthenolide, suppresses the growth and stemness of liver cancer stem cells with metabolic vulnerability. Cell Death Discovery. (2023) 9:1640. doi: 10.1038/s41420-023-01640-6 PMC10518014 37741815

[58] Tan M Mosaoa R Graham GT Kasprzyk-Pawelec A Gadre S Parasido E. Inhibition of the mitochondrial citrate carrier, Slc25a1, reverts steatosis, glucose intolerance, and inflammation in preclinical models of NAFLD/NASH. Cell Death Differ. (2020) 27(7):2143–57. doi: 10.1038/s41418-020-0491-6 PMC7308387 31959914

[59] Han Q Chen C-A Yang W Liang D Lv H-W Lv G-S. ATP-citrate lyase regulates stemness and metastasis in hepatocellular carcinoma via the Wnt/β-catenin signaling pathway. Hepatobiliary Pancreat Dis. (2021) 20(2):147–56. doi: 10.1016/j.hbpd.2020.05.010 33129711

[60] Sun H Wang F Huang Y Wang J Zhang L Shen Y. Targeted inhibition of ACLY expression to reverse the resistance of sorafenib in hepatocellular carcinoma. J Cancer. (2022) 13:951–64. doi: 10.7150/jca.52778 PMC8824901 35154461

[61] Schwenk RW Holloway GP Luiken JJFP Bonen A Glatz JFC. Fatty acid transport across the cell membrane: Regulation by fatty acid transporters. Prostaglandins Leukotrienes Essential Fatty Acids (PLEFA). (2010) 82:149–54. doi: 10.1016/j.plefa.2010.02.029 20206486

[62] Tao L Ding X Yan L Xu G Zhang P Ji A. CD36 accelerates the progression of hepatocellular carcinoma by promoting FAs absorption. Med Oncol. (2022) 39:202. doi: 10.1007/s12032-022-01808-7 36175596

[63] Menendez JA. Fine-tuning the lipogenic/lipolytic balance to optimize the metabolic requirements of cancer cell growth: Molecular mechanisms and therapeutic perspectives. Biochim Biophys Acta (BBA) - Mol Cell Biol Lipids. (2010) 1801:381–91. doi: 10.1016/j.bbalip.2009.09.005 19782152

[64] Petan T Jarc E Jusović M. Lipid droplets in cancer: guardians of fat in a stressful world. Molecules. (2018) 23:1941. doi: 10.3390/molecules23081941 30081476 PMC6222695

[65] Hao L Guo Y Peng Q Zhang Z Ji J Liu Y. Dihydroartemisinin reduced lipid droplet deposition by YAP1 to promote the anti-PD-1 effect in hepatocellular carcinoma. Phytomedicine. (2022) 96:153913. doi: 10.1016/j.phymed.2021.153913 35026515

[66] Pavlova NN Hui S Ghergurovich JM Fan J Intlekofer AM White RM. As extracellular glutamine levels decline, asparagine becomes an essential amino acid. Cell Metab. (2018) 27:428–438.e5. doi: 10.1016/j.cmet.2017.12.006 29337136 PMC5803449

[67] Krall AS Xu S Graeber TG Braas D Christofk HR. Asparagine promotes cancer cell proliferation through use as an amino acid exchange factor. Nat Commun. (2016) 7:11457. doi: 10.1038/ncomms11457 27126896 PMC4855534

[68] Zhou Q Li L Sha F Lei Y Tian X Chen L. PTTG1 reprograms asparagine metabolism to promote hepatocellular carcinoma progression. Cancer Res. (2023) 83:2372–86. doi: 10.1158/0008-5472.CAN-22-3561 37159932

[69] Qiu H Shao N Liu J Zhao J Chen C Li Q. Amino acid metabolism in tumor: New shine in the fog? Clin Nutr. (2023) 42:1521–30. doi: 10.1016/j.clnu.2023.06.011 37321900

[70] Safrhansova L Hlozkova K Starkova J. Targeting amino acid metabolism in cancer. Int Rev Cell Mol Biol. (2022) 373:37–79. doi: 10.1016/bs.ircmb.2022.08.001 36283767

[71] Mossmann D Müller C Park S Ryback B Colombi M Ritter N. Arginine reprograms metabolism in liver cancer via RBM39. Cell. (2023) 186:5068–5083.e23. doi: 10.1016/j.cell.2023.09.011 37804830 PMC10642370

[72] Pascale RM Feo CF Calvisi DF Feo F. Deregulation of methionine metabolism as determinant of progression and prognosis of hepatocellular carcinoma. Transl Gastroenterol Hepatol. (2018) 3:36–6. doi: 10.21037/tgh.2018.06.04 PMC6044036 30050996

[73] Li F Liu P Mi W Li L Anderson NM Lesner NP. Blocking methionine catabolism induces senescence and confers vulnerability to GSK3 inhibition in liver cancer. Nat Cancer. (2024) 5:131–46. doi: 10.1038/s43018-023-00671-3 PMC11277537 38168934

[74] Yamamoto J Inubushi S Han Q Tashiro Y Sugisawa N Hamada K. Linkage of methionine addiction, histone lysine hypermethylation, and Malignancy. iScience. (2022) 25(4):104162. doi: 10.1016/j.isci.2022.104162 35434545 PMC9010622

[75] Conrad M Sato H. The oxidative stress-inducible cystine/glutamate antiporter, system x c –: cystine supplier and beyond. Amino Acids. (2012) 42:231–46. doi: 10.1007/s00726-011-0867-5 21409388

[76] Fujii J Homma T Kobayashi S. Ferroptosis caused by cysteine insufficiency and oxidative insult. Free Radical Res. (2020) 54:969–80. doi: 10.1080/10715762.2019.1666983 31505959

[77] Hu X He Y Han Z Liu W Liu D Zhang X. PNO1 inhibits autophagy-mediated ferroptosis by GSH metabolic reprogramming in hepatocellular carcinoma. Cell Death Dis. (2022) 13:1010. doi: 10.1038/s41419-022-05448-7 36446769 PMC9709074

[78] Ding Y Wang X Lu S Lai A Xie B He X. BCAT1, as a prognostic factor for HCC, can promote the development of liver cancer through activation of the AKT signaling pathway and EMT. J Mol Histol. (2023) 54:25–39. doi: 10.1007/s10735-022-10108-3 36344754

[79] Zou H Liao M Xu W Yao R Liao W. Data mining of the expression and regulatory role of BCAT1 in hepatocellular carcinoma. Oncol Lett. (2019) 18(6):5879–88. doi: 10.3892/ol.2019.10932 PMC6865021 31788061

[80] Missiaen R Anderson NM Kim LC Nance B Burrows M Skuli N. GCN2 inhibition sensitizes arginine-deprived hepatocellular carcinoma cells to senolytic treatment. Cell Metab. (2022) 34:1151–1167.e7. doi: 10.1016/j.cmet.2022.06.010 35839757 PMC9357184

[81] Hu X Chen Z Wang Z Xiao Q. Cancer evolution: Special focus on the immune aspect of cancer. Semin Cancer Biol. (2022) 86:420–35. doi: 10.1016/j.semcancer.2022.05.006 35589072

[82] Bergers G Fendt S-M. The metabolism of cancer cells during metastasis. Nat Rev Cancer. (2021) 21:162–80. doi: 10.1038/s41568-020-00320-2 PMC8733955 33462499

[83] Yuneva MO Fan TWM Allen TD Higashi RM Ferraris DV Tsukamoto T. The metabolic profile of tumors depends on both the responsible genetic lesion and tissue type. Cell Metab. (2012) 15:157–70. doi: 10.1016/j.cmet.2011.12.015 PMC3282107 22326218

[84] Hastak K Paul RK Agarwal MK Thakur VS Amin ARMR Agrawal S. DNA synthesis from unbalanced nucleotide pools causes limited DNA damage that triggers ATR-CHK1-dependent p53 activation. Proc Natl Acad Sci U.S.A. (2008) 105:6314–9. doi: 10.1073/pnas.0802080105 PMC2359797 18434539

[85] Wong T-L Ng K-Y Tan KV Chan L-H Zhou L Che N. CRAF methylation by PRMT6 regulates aerobic glycolysis-driven hepatocarcinogenesis via ERK-dependent PKM2 nuclear relocalization and activation. Hepatology. (2020) 71:1279–96. doi: 10.1002/hep.30923 31469916

[86] Senni N Savall M Cabrerizo Granados D Alves-Guerra M-C Sartor C Lagoutte I. [amp]]beta;-catenin-activated hepatocellular carcinomas are addicted to fatty acids. Gut. (2019) 68:322–34. doi: 10.1136/gutjnl-2017-315448 29650531

[87] Chen C-L Uthaya Kumar DB Punj V Xu J Sher L Tahara SM. NANOG metabolically reprograms tumor-initiating stem-like cells through tumorigenic changes in oxidative phosphorylation and fatty acid metabolism. Cell Metab. (2016) 23:206–19. doi: 10.1016/j.cmet.2015.12.004 PMC4715587 26724859

[88] Xia S Pan Y Liang Y Xu J Cai X. The microenvironmental and metabolic aspects of sorafenib resistance in hepatocellular carcinoma. EBioMedicine. (2020) 51:102610. doi: 10.1016/j.ebiom.2019.102610 31918403 PMC7000339

[89] Casadei-Gardini A Del Coco L Marisi G Conti F Rovesti G Ulivi P. 1H-NMR based serum metabolomics highlights different specific biomarkers between early and advanced hepatocellular carcinoma stages. Cancers. (2020) 12:241. doi: 10.3390/cancers12010241 31963766 PMC7016798

[90] Bidkhori G Benfeitas R Klevstig M Zhang C Nielsen J Uhlen M. Metabolic network-based stratification of hepatocellular carcinoma reveals three distinct tumor subtypes. Proc Natl Acad Sci USA. (2018) 115:E11874–83. doi: 10.1073/pnas.1807305115 PMC6294939 30482855

[91] Daniels NJ Hershberger CE Kerosky M Wehrle CJ Raj R Aykun N. Biomarker discovery in liver disease using untargeted metabolomics in plasma and saliva. Int J Mol Sci. (2024) 25:10144. doi: 10.3390/ijms251810144 39337628 PMC11432510

[92] Guo D-Z Zhang X Zhang S-Q Zhang S-Y Zhang X-Y Yan J-Y. Single-cell tumor heterogeneity landscape of hepatocellular carcinoma: Unraveling the pro-metastatic subtype and its interaction loop with fibroblasts. Mol Cancer. (2024) 23:157. doi: 10.1186/s12943-024-02062-3 39095854 PMC11295380

[93] Liu Y Liang G Xu H Dong W Dong Z Qiu Z. Tumors exploit FTO-mediated regulation of glycolytic metabolism to evade immune surveillance. Cell Metab. (2021) 33:1221–1233.e11. doi: 10.1016/j.cmet.2021.04.001 33910046

[94] Kamm DR McCommis KS. Hepatic stellate cells in physiology and pathology. J Physiol. (2022) 600:1825–37. doi: 10.1113/JP281061 PMC9012702 35307840

[95] Molecular pathogenesis of hepatic fibrosis and current therapeutic approaches . Available online at (Accessed March 4, 2024). 10.1016/j.cbi.2011.07.001 PMC3171510 21803030

[96] Mattos ÂZ Debes JD Vogel A Arrese M Revelo X Pase THS. Non-alcoholic fatty liver disease-related hepatocellular carcinoma: Is there a role for immunotherapy? World J Gastroenterol. (2022) 28:3595–607. doi: 10.3748/wjg.v28.i28.3595 PMC9372815 36161041

[97] Lin Y Xu J Lan H. Tumor-associated macrophages in tumor metastasis: biological roles and clinical therapeutic applications. J Hematol Oncol. (2019) 12:76. doi: 10.1186/s13045-019-0760-3 31300030 PMC6626377

[98] Liu P Kong L Liu Y Li G Xie J Lu X. A key driver to promote HCC: Cellular crosstalk in tumor microenvironment. Front Oncol. (2023) 13:1135122. doi: 10.3389/fonc.2023.1135122 37007125 PMC10050394

[99] Sung PS. Crosstalk between tumor-associated macrophages and neighboring cells in hepatocellular carcinoma. Clin Mol Hepatol. (2022) 28:333–50. doi: 10.3350/cmh.2021.0308 PMC9293612 34665953

[100] Hu C Pang B Lin G Zhen Y Yi H. Energy metabolism manipulates the fate and function of tumour myeloid-derived suppressor cells. Br J Cancer. (2020) 122:23–9. doi: 10.1038/s41416-019-0644-x PMC6964679 31819182

[101] Baglieri J Brenner DA Kisseleva T. The role of fibrosis and liver-associated fibroblasts in the pathogenesis of hepatocellular carcinoma. Int J Mol Sci. (2019) 20:1723. doi: 10.3390/ijms20071723 30959975 PMC6479943

[102] Gao J Li Z Lu Q Zhong J Pan L Feng C. Single-cell RNA sequencing reveals cell subpopulations in the tumor microenvironment contributing to hepatocellular carcinoma. Front Cell Dev Biol. (2023) 11:1194199. doi: 10.3389/fcell.2023.1194199 37333982 PMC10272598

[103] Song J Ge Z Yang X Luo Q Wang C You H. Hepatic stellate cells activated by acidic tumor microenvironment promote the metastasis of hepatocellular carcinoma via osteopontin. Cancer Lett. (2015) 356:713–20. doi: 10.1016/j.canlet.2014.10.021 25449435

[104] Loh J-J Li T-W Zhou L Wong T-L Liu X Ma VWS. FSTL1 secreted by activated fibroblasts promotes hepatocellular carcinoma metastasis and stemness. Cancer Res. (2021) 81:5692–705. doi: 10.1158/0008-5472.CAN-20-4226 34551961

[105] Qu C He L Yao N Li J Jiang Y Li B. Myofibroblast-specific msi2 knockout inhibits HCC progression in a mouse model. Hepatology. (2021) 74:458–73. doi: 10.1002/hep.31754 33609283

[106] Wang F Chen L Kong D Zhang X Xia S Liang B. Canonical Wnt signaling promotes HSC glycolysis and liver fibrosis through an LDH-A/HIF-1α transcriptional complex. Hepatology. (2024) 79:606–23. doi: 10.1097/HEP.0000000000000569 PMC10871634 37733267

[107] Chen Y Choi SS Michelotti GA Chan IS Swiderska-Syn M Karaca GF. Hedgehog controls hepatic stellate cell fate by regulating metabolism. Gastroenterology. (2012) 143:1319–1329.e11. doi: 10.1053/j.gastro.2012.07.115 22885334 PMC3480563

[108] Trivedi P Wang S Friedman SL. The power of plasticity-metabolic regulation of hepatic stellate cells. Cell Metabolism. (2021) 33(2):242–56. doi: 10.1016/j.cmet.2020.10.026 PMC7858232 33232666

[109] Du K Hyun J Premont RT Choi SS Michelotti GA Swiderska-Syn M. Hedgehog-YAP signaling pathway regulates glutaminolysis to control activation of hepatic stellate cells. Gastroenterology. (2018) 154:1465–1479.e13. doi: 10.1053/j.gastro.2017.12.022 29305935 PMC5880682

[110] Mallikarjuna P Zhou Y Landström M. The synergistic cooperation between TGF-β and hypoxia in cancer and fibrosis. Biomolecules. (2022) 12:635. doi: 10.3390/biom12050635 35625561 PMC9138354

[111] Birbrair A ed. Tumor Microenvironment: Non-Hematopoietic Cells. Cham: Springer International Publishing (2020). doi: 10.1007/978-3-030-37184-5

[112] Li J Yan Y Ang L Li X Liu C Sun B. Extracellular vesicles-derived OncomiRs mediate communication between cancer cells and cancer-associated hepatic stellate cells in hepatocellular carcinoma microenvironment. Carcinogenesis. (2020) 41:223–34. doi: 10.1093/carcin/bgz096 31140556

[113] Cho Y Cho EJ Lee J-H Yu SJ Kim YJ Kim CY. Hypoxia enhances tumor-stroma crosstalk that drives the progression of hepatocellular carcinoma. Dig Dis Sci. (2016) 61:2568–77. doi: 10.1007/s10620-016-4158-6 27074919

[114] Khanal S Liu Y Bamidele AO Wixom AQ Washington AM Jalan-Sakrikar N. Glycolysis in hepatic stellate cells coordinates fibrogenic extracellular vesicle release spatially to amplify liver fibrosis. Sci Adv. (2024) 10:eadn5228. doi: 10.1126/sciadv.adn5228 38941469 PMC11212729

[115] Wang Y Huang Y Guan F Xiao Y Deng J Chen H. Hypoxia-inducible factor-1alpha and MAPK co-regulate activation of hepatic stellate cells upon hypoxia stimulation. PloS One. (2013) 8:e74051. doi: 10.1371/journal.pone.0074051 24040163 PMC3769364

[116] Trillos-Almanza MC Aguilar MM Buist-Homan M Bomer N Gomez KA de Meijer VE. Branched-chain amino acids and their metabolites decrease human and rat hepatic stellate cell activation. Mol Biol Rep. (2024) 51:1116. doi: 10.1007/s11033-024-10027-4 39495311 PMC11534903

[117] Zhou Y Ren H Dai B Li J Shang L Huang J. Hepatocellular carcinoma-derived exosomal miRNA-21 contributes to tumor progression by converting hepatocyte stellate cells to cancer-associated fibroblasts. J Exp Clin Canc Res. (2018) 37:324. doi: 10.1186/s13046-018-0965-2 PMC6307162 30591064

[118] Yu Y Li Y Zhou L Cheng X Gong Z. Hepatic stellate cells promote hepatocellular carcinoma development by regulating histone lactylation: Novel insights from single-cell RNA sequencing and spatial transcriptomics analyses. Cancer Lett. (2024) 604:217243. doi: 10.1016/j.canlet.2024.217243 39260669

[119] Sun Y Zhang H Li Y Han J. Abnormal metabolism in hepatic stellate cells: Pandora’s box of MAFLD related hepatocellular carcinoma. Biochim Biophys Acta (BBA) - Rev Cancer. (2024) 1879:189086. doi: 10.1016/j.bbcan.2024.189086 38342420

[120] Sahai E Astsaturov I Cukierman E DeNardo DG Egeblad M Evans RM. A framework for advancing our understanding of cancer-associated fibroblasts. Nat Rev Cancer. (2020) 20:174–86. doi: 10.1038/s41568-019-0238-1 PMC7046529 31980749

[121] Eun JW Yoon JH Ahn HR Kim S Kim YB Lim SB. Cancer-associated fibroblast-derived secreted phosphoprotein 1 contributes to resistance of hepatocellular carcinoma to sorafenib and lenvatinib. Cancer Commun. (2023) 43:455–79. doi: 10.1002/cac2.12414 PMC10091107 36919193

[122] Liang L Li W Li X Jin X Liao Q Li Y. [amp]]lsquo;Reverse Warburg effect’ of cancer−associated fibroblasts (Review). Int J Oncol. (2022) 60:67. doi: 10.3892/ijo.2022.5357 35425996

[123] Pavlides S Whitaker-Menezes D Castello-Cros R Flomenberg N Witkiewicz AK Frank PG. The reverse Warburg effect: Aerobic glycolysis in cancer associated fibroblasts and the tumor stroma. Cell Cycle. (2009) 8:3984–4001. doi: 10.4161/cc.8.23.10238 19923890

[124] Li Z Sun C Qin Z. Metabolic reprogramming of cancer-associated fibroblasts and its effect on cancer cell reprogramming. Theranostics. (2021) 11:8322–36. doi: 10.7150/thno.62378 PMC8343997 34373744

[125] Grassian AR Coloff JL Brugge JS. Extracellular matrix regulation of metabolism and implications for tumorigenesis. Cold Spring Harb Symp Quant Biol. (2011) 76:313–24. doi: 10.1101/sqb.2011.76.010967 22105806

[126] Wang H Liu F Wu X Zhu G Tang Z Qu W. Cancer-associated fibroblasts contributed to hepatocellular carcinoma recurrence and metastasis via CD36-mediated fatty-acid metabolic reprogramming. Exp Cell Res. (2024) 435:113947. doi: 10.1016/j.yexcr.2024.113947 38301989

[127] Gong J Lin Y Zhang H Liu C Cheng Z Yang X. Reprogramming of lipid metabolism in cancer-associated fibroblasts potentiates migration of colorectal cancer cells. Cell Death Dis. (2020) 11:267. doi: 10.1038/s41419-020-2434-z 32327627 PMC7181758

[128] Peng S Li Y Huang M Tang G Xie Y Chen D. Metabolomics reveals that CAF-derived lipids promote colorectal cancer peritoneal metastasis by enhancing membrane fluidity. Int J Biol Sci. (2022) 18:1912–32. doi: 10.7150/ijbs.68484 PMC8935219 35342344

[129] Nath A Li I Roberts LR Chan C. Elevated free fatty acid uptake via CD36 promotes epithelial-mesenchymal transition in hepatocellular carcinoma. Sci Rep. (2015) 5:14752. doi: 10.1038/srep14752 26424075 PMC4589791

[130] Zhu G-Q Tang Z Huang R Qu W-F Fang Y Yang R. CD36+ cancer-associated fibroblasts provide immunosuppressive microenvironment for hepatocellular carcinoma via secretion of macrophage migration inhibitory factor. Cell Discovery. (2023) 9:25. doi: 10.1038/s41421-023-00529-z 36878933 PMC9988869

[131] Mazzocca A Dituri F Lupo L Quaranta M Antonaci S Giannelli G. Tumor-secreted lysophostatidic acid accelerates hepatocellular carcinoma progression by promoting differentiation of peritumoral fibroblasts in myofibroblasts. Hepatology. (2011) 54:920–30. doi: 10.1002/hep.24485 21674557

[132] Mestre-Farrera A Bruch-Oms M Peña R Rodríguez-Morató J Alba-Castellón L Comerma L. Glutamine-directed migration of cancer-activated fibroblasts facilitates epithelial tumor invasion. Cancer Res. (2021) 81:438–51. doi: 10.1158/0008-5472.CAN-20-0622 33229340

[133] Santi A Caselli A Ranaldi F Paoli P Mugnaioni C Michelucci E. Cancer associated fibroblasts transfer lipids and proteins to cancer cells through cargo vesicles supporting tumor growth. Biochim Biophys Acta (BBA) - Mol Cell Res. (2015) 1853:3211–23. doi: 10.1016/j.bbamcr.2015.09.013 26384873

[134] Lu L Huang J Mo J Da X Li Q Fan M. Exosomal lncRNA TUG1 from cancer-associated fibroblasts promotes liver cancer cell migration, invasion, and glycolysis by regulating the miR-524-5p/SIX1 axis. Cell Mol Biol Lett. (2022) 27:17. doi: 10.1186/s11658-022-00309-9 35193488 PMC8903597

[135] Liu J Chen S Wang W Ning B-F Chen F Shen W. Cancer-associated fibroblasts promote hepatocellular carcinoma metastasis through chemokine-activated hedgehog and TGF-β pathways. Cancer Lett. (2016) 379:49–59. doi: 10.1016/j.canlet.2016.05.022 27216982

[136] Yuan Q Zhang J Liu Y Chen H Liu H Wang J. MyD88 in myofibroblasts regulates aerobic glycolysis-driven hepatocarcinogenesis via ERK-dependent PKM2 nuclear relocalization and activation. J Pathol. (2022) 256(4):414–26. doi: 10.1002/path.5856 34927243

[137] Xu H Zhao J Li J Zhu Z Cui Z Liu R. Cancer associated fibroblast-derived CCL5 promotes hepatocellular carcinoma metastasis through activating HIF1α/ZEB1 axis. Cell Death Dis. (2022) 13:478. doi: 10.1038/s41419-022-04935-1 35589690 PMC9119971

[138] Karta J Bossicard Y Kotzamanis K Dolznig H Letellier E. Mapping the metabolic networks of tumor cells and cancer-associated fibroblasts. Cells. (2021) 10:304. doi: 10.3390/cells10020304 33540679 PMC7912987

[139] Liu Q-P Luo Q Deng B Ju Y Song G-B. Stiffer matrix accelerates migration of hepatocellular carcinoma cells through enhanced aerobic glycolysis via the MAPK-YAP signaling. Cancers. (2020) 12:490. doi: 10.3390/cancers12020490 32093118 PMC7072284

[140] Bertero T Oldham WM Grasset EM Bourget I Boulter E Pisano S. Tumor-stroma mechanics coordinate amino acid availability to sustain tumor growth and Malignancy. Cell Metab. (2019) 29:124–140.e10. doi: 10.1016/j.cmet.2018.09.012 30293773 PMC6432652

[141] Wang Y Wang D Yang L Zhang Y. Metabolic reprogramming in the immunosuppression of tumor-associated macrophages. Chin Med J (engl). (2022) 135:2405–16. doi: 10.1097/CM9.0000000000002426 PMC9945195 36385099

[142] Corbet C Feron O. Tumour acidosis: from the passenger to the driver’s seat. Nat Rev Cancer. (2017) 17:577–93. doi: 10.1038/nrc.2017.77 28912578

[143] Lu H Dalgard CL Mohyeldin A McFate T Tait AS Verma A. Reversible inactivation of HIF-1 prolyl hydroxylases allows cell metabolism to control basal HIF-1. J Biol Chem. (2005) 280:41928–39. doi: 10.1074/jbc.M508718200 16223732

[144] Colegio OR Chu N-Q Szabo AL Chu T Rhebergen AM Jairam V. Functional polarization of tumour-associated macrophages by tumour-derived lactic acid. Nature. (2014) 513:559–63. doi: 10.1038/nature13490 PMC4301845 25043024

[145] Stockmann C Doedens A Weidemann A Zhang N Takeda N Greenberg JI. Deletion of vascular endothelial growth factor in myeloid cells accelerates tumorigenesis. Nature. (2008) 456:814–8. doi: 10.1038/nature07445 PMC3103772 18997773

[146] Li J DeNicola GM Ruffell B. Metabolism in tumor-associated macrophages. Int Rev Cell Mol Biol. (2022) 373:65–100. doi: 10.1016/bs.ircmb.2022.01.004 PMC9094395 35461660

[147] Zhao X Li K Chen M Liu L. Metabolic codependencies in the tumor microenvironment and gastric cancer: Difficulties and opportunities. Biomed Pharmacother. (2023) 162:114601. doi: 10.1016/j.biopha.2023.114601 36989719

[148] Feng R Morine Y Ikemoto T Imura S Iwahashi S Saito Y. Nrf2 activation drive macrophages polarization and cancer cell epithelial-mesenchymal transition during interaction. Cell Commun Signal. (2018) 16:54. doi: 10.1186/s12964-018-0262-x 30180849 PMC6122794

[149] Linton SS Abraham T Liao J Clawson GA Butler PJ Fox T. Tumor-promoting effects of pancreatic cancer cell exosomes on THP-1-derived macrophages. PloS One. (2018) 13:e0206759. doi: 10.1371/journal.pone.0206759 30383833 PMC6211741

[150] Liu B Qu L Yan S. Cyclooxygenase-2 promotes tumor growth and suppresses tumor immunity. Cancer Cell Int. (2015) 15:106. doi: 10.1186/s12935-015-0260-7 26549987 PMC4635545

[151] Antonioli L Blandizzi C Pacher P Haskó G. Immunity, inflammation and cancer: a leading role for adenosine. Nat Rev Cancer. (2013) 13:842–57. doi: 10.1038/nrc3613 24226193

[152] Khalid M Brisson L Tariq M Hao Y Guibon R Fromont G. Carcinoma-specific expression of P2Y11 receptor and its contribution in ATP-induced purinergic signalling and cell migration in human hepatocellular carcinoma cells. Oncotarget. (2017) 8:37278–90. doi: 10.18632/oncotarget.16191 PMC5514908 28418839

[153] He Y Xu H Liu Y Kempa S Vechiatto C Schmidt R. The effects of hypoxia on the immune-metabolic interplay in liver cancer. Biomolecules. (2024) 14:1024. doi: 10.3390/biom14081024 39199411 PMC11352590

[154] Wang J Wang Y Chu Y Li Z Yu X Huang Z. Tumor-derived adenosine promotes macrophage proliferation in human hepatocellular carcinoma. J Hepatol. (2021) 74:627–37. doi: 10.1016/j.jhep.2020.10.021 33137360

[155] Yang M McKay D Pollard JW Lewis CE. Diverse functions of macrophages in different tumor microenvironments. Cancer Res. (2018) 78:5492–503. doi: 10.1158/0008-5472.CAN-18-1367 PMC6171744 30206177

[156] Wang F Zhang S Vuckovic I Jeon R Lerman A Folmes CD. Glycolytic stimulation is not a requirement for M2 macrophage differentiation. Cell Metab. (2018) 28:463–475.e4. doi: 10.1016/j.cmet.2018.08.012 30184486 PMC6449248

[157] Chen D Zhang X Li Z Zhu B. Metabolic regulatory crosstalk between tumor microenvironment and tumor-associated macrophages. Theranostics. (2021) 11:1016–30. doi: 10.7150/thno.51777 PMC7738889 33391518

[158] Wu K Kryczek I Chen L Zou W. Kupffer cell suppression of CD8+ T cells in human hepatocellular carcinoma is mediated by B7-H1/programmed death-1 interactions. Cancer Res. (2009) 69(20):8067–75. doi: 10.1158/0008-5472.CAN-09-0901 PMC4397483 19826049

[159] Wang R Dillon CP Shi LZ Milasta S Carter R Finkelstein D. The transcription factor myc controls metabolic reprogramming upon T lymphocyte activation. Immunity. (2011) 35:871–82. doi: 10.1016/j.immuni.2011.09.021 PMC3248798 22195744

[160] Chapman NM Boothby MR Chi H. Metabolic coordination of T cell quiescence and activation. Nat Rev Immunol. (2020) 20:55–70. doi: 10.1038/s41577-019-0203-y 31406325

[161] Boomer JS Green JM. An enigmatic tail of CD28 signaling. Cold Spring Harbor Perspect Biol. (2010) 2:a002436–a002436. doi: 10.1101/cshperspect.a002436 PMC2908766 20534709

[162] Sena LA Li S Jairaman A Prakriya M Ezponda T Hildeman DA. Mitochondria are required for antigen-specific T cell activation through reactive oxygen species signaling. Immunity. (2013) 38:225–36. doi: 10.1016/j.immuni.2012.10.020 PMC3582741 23415911

[163] Sugiura A Rathmell JC. Metabolic barriers to T cell function in tumors. J Immunol. (2018) 200:400–7. doi: 10.4049/jimmunol.1701041 PMC5777533 29311381

[164] Michalek RD Gerriets VA Jacobs SR Macintyre AN MacIver NJ Mason EF. Cutting edge: distinct glycolytic and lipid oxidative metabolic programs are essential for effector and regulatory CD4+ T cell subsets. J Immunol. (2011) 186:3299–303. doi: 10.4049/jimmunol.1003613 PMC3198034 21317389

[165] Hu B Yu M Ma X Sun J Liu C Wang C. IFNα Potentiates anti–PD-1 efficacy by remodeling glucose metabolism in the hepatocellular carcinoma microenvironment. Cancer Discovery. (2022) 12:1718–41. doi: 10.1158/2159-8290.CD-21-1022 35412588

[166] Watson MJ Vignali PDA Mullett SJ Overacre-Delgoffe AE Peralta RM Grebinoski S. Metabolic support of tumour-infiltrating regulatory T cells by lactic acid. Nature. (2021) 591:645–51. doi: 10.1038/s41586-020-03045-2 PMC7990682 33589820

[167] Zhang H Li S Wang D Liu S Xiao T Gu W. Metabolic reprogramming and immune evasion: The interplay in the tumor microenvironment. biomark Res. (2024) 12:96. doi: 10.1186/s40364-024-00646-1 39227970 PMC11373140

[168] Kumagai S Koyama S Itahashi K Tanegashima T Lin Y Togashi Y. Lactic acid promotes PD-1 expression in regulatory T cells in highly glycolytic tumor microenvironments. Cancer Cell. (2022) 40:201–218.e9. doi: 10.1016/j.ccell.2022.01.001 35090594

[169] Schafer JR Salzillo TC Chakravarti N Kararoudi MN Trikha P Foltz JA. Education-dependent activation of glycolysis promotes the cytolytic potency of licensed human natural killer cells. J Allergy Clin Immunol. (2019) 143:346–358.e6. doi: 10.1016/j.jaci.2018.06.047 30096390

[170] Devillier R Chrétien A-S Pagliardini T Salem N Blaise D Olive D. Mechanisms of NK cell dysfunction in the tumor microenvironment and current clinical approaches to harness NK cell potential for immunotherapy. J Leukocyte Biol. (2021) 109:1071–88. doi: 10.1002/JLB.5MR0920-198RR 32991746

[171] Husain Z Huang Y Seth P Sukhatme VP. Tumor-derived lactate modifies antitumor immune response: effect on myeloid-derived suppressor cells and NK cells. J Immunol. (2013) 191:1486–95. doi: 10.4049/jimmunol.1202702 23817426

[172] Geiger R Rieckmann JC Wolf T Basso C Feng Y Fuhrer T. L-arginine modulates T cell metabolism and enhances survival and anti-tumor activity. Cell. (2016) 167:829–842.e13. doi: 10.1016/j.cell.2016.09.031 27745970 PMC5075284

[173] Mills C. M1 and M2 macrophages: oracles of health and disease. Crit Rev Immunol. (2012) 32:463–88. doi: 10.1615/CritRevImmunol.v32.i6.10 23428224

[174] Di Virgilio F Sarti AC Falzoni S De Marchi E Adinolfi E. Extracellular ATP and P2 purinergic signalling in the tumour microenvironment. Nat Rev Cancer. (2018) 18:601–18. doi: 10.1038/s41568-018-0037-0 30006588

[175] Tang W Zhou J Yang W Feng Y Wu H Mok MTS. Aberrant cholesterol metabolic signaling impairs antitumor immunosurveillance through natural killer T cell dysfunction in obese liver. Cell Mol Immunol. (2022) 19:834–47. doi: 10.1038/s41423-022-00872-3 PMC9243114 35595819

[176] Kim H-J Cantor H. CD4 T-cell subsets and tumor immunity: The helpful and the not-so-helpful. Cancer Immunol Res. (2014) 2:91–8. doi: 10.1158/2326-6066.CIR-13-0216 24778273

[177] Ma C Kesarwala AH Eggert T Medina-Echeverz J Kleiner DE Jin P. NAFLD causes selective CD4(+) T lymphocyte loss and promotes hepatocarcinogenesis. Nature. (2016) 531:253–7. doi: 10.1038/nature16969 PMC4786464 26934227

[178] Brown ZJ Fu Q Ma C Kruhlak M Zhang H Luo J. Carnitine palmitoyltransferase gene upregulation by linoleic acid induces CD4+ T cell apoptosis promoting HCC development. Cell Death Dis. (2018) 9:620. doi: 10.1038/s41419-018-0687-6 29795111 PMC5966464

[179] Ma K Chu J Liu Y Sun L Zhou S Li X. Hepatocellular carcinoma LINC01116 outcompetes T cells for linoleic acid and accelerates tumor progression. Adv Sci (Weinheim Baden-Wurttemberg Germany). (2024) 11:e2400676. doi: 10.1002/advs.202400676 PMC11151013 38460179

[180] Jian S-L Chen W-W Su Y-C Su Y-W Chuang T-H Hsu S-C. Glycolysis regulates the expansion of myeloid-derived suppressor cells in tumor-bearing hosts through prevention of ROS-mediated apoptosis. Cell Death Dis. (2017) 8:e2779–9. doi: 10.1038/cddis.2017.192 PMC5520713 28492541

[181] Hossain F Al-Khami AA Wyczechowska D Hernandez C Zheng L Reiss K. Inhibition of fatty acid oxidation modulates immunosuppressive functions of myeloid-derived suppressor cells and enhances cancer therapies. Cancer Immunol Res. (2015) 3:1236–47. doi: 10.1158/2326-6066.CIR-15-0036 PMC4636942 26025381

[182] Grzywa TM Sosnowska A Matryba P Rydzynska Z Jasinski M Nowis D. Myeloid cell-derived arginase in cancer immune response. Front Immunol. (2020) 11:938. doi: 10.3389/fimmu.2020.00938 32499785 PMC7242730

[183] Cimen Bozkus C Elzey BD Crist SA Ellies LG Ratliff TL. Expression of cationic amino acid transporter 2 is required for myeloid-derived suppressor cell–mediated control of T cell immunity. J Immunol. (2015) 195:5237–50. doi: 10.4049/jimmunol.1500959 PMC4655170 26491198

[184] Sica A Strauss L Consonni FM Travelli C Genazzani A Porta C. Metabolic regulation of suppressive myeloid cells in cancer. Cytokine Growth Factor Rev. (2017) 35:27–35. doi: 10.1016/j.cytogfr.2017.05.002 28499577

[185] Li X Luo X Chen S Chen J Deng X Zhong J. All-trans-retinoic acid inhibits hepatocellular carcinoma progression by targeting myeloid-derived suppressor cells and inhibiting angiogenesis. Int Immunopharmacol. (2023) 121:110413. doi: 10.1016/j.intimp.2023.110413 37301119

[186] Srivastava MK Sinha P Clements VK Rodriguez P Ostrand-Rosenberg S. Myeloid-derived suppressor cells inhibit T-cell activation by depleting cystine and cysteine. Cancer Res. (2010) 70:68–77. doi: 10.1158/0008-5472.CAN-09-2587 20028852 PMC2805057

[187] Wang Y Jia A Bi Y Wang Y Liu G. Metabolic regulation of myeloid-derived suppressor cell function in cancer. Cells. (2020) 9:1011. doi: 10.3390/cells9041011 32325683 PMC7226088

[188] Mei Y Zhu Y Yong KSM Hanafi ZB Gong H Liu Y. IL-37 dampens immunosuppressive functions of MDSCs via metabolic reprogramming in the tumor microenvironment. Cell Rep. (2024) 43:113835. doi: 10.1016/j.celrep.2024.113835 38412100

[189] Cho YY Yu SJ Lee HW Kim DY Kang W Paik Y-H. Clinical characteristics of long-term survivors after sorafenib treatment for unresectable hepatocellular carcinoma: A korean national multicenter retrospective cohort study. J Hepatocell Carcinoma. (2021) 8:613–23. doi: 10.2147/JHC.S304439 PMC8219232 34169044

[190] Price GS Page RL Riviere JE Cline JM Thrall DE. Pharmacokinetics and toxicity of oral and intravenous lonidamine in dogs. Cancer Chemother Pharmacol. (1996) 38:129–35. doi: 10.1007/s002800050460 8616902

[191] Tomizawa M Shinozaki F Motoyoshi Y Sugiyama T Yamamoto S Ishige N. 2-deoxyglucose and sorafenib synergistically suppress the proliferation and motility of hepatocellular carcinoma cells. Oncol Lett. (2017) 13:800–4. doi: 10.3892/ol.2016.5510 PMC5351389 28356961

[192] Wang L Yang Q Peng S Liu X. The combination of the glycolysis inhibitor 2-DG and sorafenib can be effective against sorafenib-tolerant persister cancer cells. OncoTargets Ther. (2019) 12:5359–73. doi: 10.2147/OTT.S212465 PMC6635829 31371980

[193] Uludağ D Bay S Sucu BO Şavluğ İpek Ö Mohr T Güzel M. Potential of novel methyl jasmonate analogs as anticancer agents to metabolically target HK-2 activity in glioblastoma cells. Front Pharmacol. (2022) 13:828400. doi: 10.3389/fphar.2022.828400 35677429 PMC9168889

[194] Rong W Wan N Zheng X Shi G Jiang C Pan K. Chrysin inhibits hepatocellular carcinoma progression through suppressing programmed death ligand 1 expression. Phytomedicine. (2022) 95:153867. doi: 10.1016/j.phymed.2021.153867 34923234

[195] Zheng D Jiang Y Qu C Yuan H Hu K He L. Pyruvate kinase M2 tetramerization protects against hepatic stellate cell activation and liver fibrosis. Am J Pathol. (2020) 190:2267–81. doi: 10.1016/j.ajpath.2020.08.002 PMC7786052 32805235

[196] Ye C Zhang J Shen J Chen R Li Q Zhao P. Attractive pickering emulsion gel loaded with oxaliplatin and lactate dehydrogenase inhibitor increases the anti-tumor effect in hepatocellular carcinoma. Chin Chem Lett. (2024) 35(10):2985–90. doi: 10.1016/j.cclet.2024.110519

[197] Xing Y-X Li M-H Tao L Ruan L-Y Hong W Chen C. Anti-cancer effects of emodin on HepG2 cells as revealed by^1^ H NMR based metabolic profiling. J Proteome Res. (2018) 17:1943–52. doi: 10.1021/acs.jproteome.8b00029 29676152

[198] Dong F He K Zhang S Song K Jiang L Hu L-P. SSRI antidepressant citalopram reverses the warburg effect to inhibit hepatocellular carcinoma by directly targeting GLUT1. Cell Rep. (2024) 43:114818. doi: 10.1016/j.celrep.2024.114818 39388353

[199] Halford S Veal GJ Wedge SR Payne GS Bacon CM Sloan P. A phase I dose-escalation study of AZD3965, an oral monocarboxylate transporter 1 inhibitor, in patients with advanced cancer. Clin Cancer Res. (2023) 29:1429–39. doi: 10.1158/1078-0432.CCR-22-2263 PMC7614436 36652553

[200] Yip-Schneider MT Wu H Njoku V Ralstin M Holcomb B Crooks PA. Effect of celecoxib and the novel anti-cancer agent, dimethylamino-parthenolide, in a developmental model of pancreatic cancer. Pancreas. (2008) 37:e45–53. doi: 10.1097/MPA.0b013e318172b4dd 18815538

[201] D’Anneo A Carlisi D Lauricella M Puleio R Martinez R Di Bella S. Parthenolide generates reactive oxygen species and autophagy in MDA-MB231 cells. A soluble parthenolide analogue inhibits tumour growth and metastasis in a xenograft model of breast cancer. Cell Death Dis. (2013) 4:e891. doi: 10.1038/cddis.2013.415 24176849 PMC3920954

[202] Velez BC Petrella CP DiSalvo KH Cheng K Kravtsov R Krasniqi D. Combined inhibition of ACLY and CDK4/6 reduces cancer cell growth and invasion. Oncol Rep. (2023) 49:32. doi: 10.3892/or.2022.8469 36562384 PMC9827262

[203] Liu JY Kuna RS Pinheiro LV Nguyen PTT Welles JE Drummond JM. Bempedoic acid suppresses diet-induced hepatic steatosis independently of ATP-citrate lyase. Cell Metab. (2024) S1550-4131(24):00410–8. doi: 10.1016/j.cmet.2024.10.014 PMC11711013 39471816

[204] Yu Y Nie Q Wang Z Di Y Chen X Ren K. Targeting acetyl-CoA carboxylase 1 for cancer therapy. Front Pharmacol. (2023) 14:1129010. doi: 10.3389/fphar.2023.1129010 36843935 PMC9950103

[205] Calle RA Amin NB Carvajal-Gonzalez S Ross TT Bergman A Aggarwal S. ACC inhibitor alone or co-administered with a DGAT2 inhibitor in patients with non-alcoholic fatty liver disease: Two parallel, placebo-controlled, randomized phase 2a trials. Nat Med. (2021) 27:1836–48. doi: 10.1038/s41591-021-01489-1 34635855

[206] Jin H Wang S Zaal EA Wang C Wu H Bosma A. A powerful drug combination strategy targeting glutamine addiction for the treatment of human liver cancer. eLife. (2020) 9:e56749. doi: 10.7554/eLife.56749 33016874 PMC7535927

[207] Chan SL Cheng PNM Liu AM Chan LL Li L Chu CM. A phase II clinical study on the efficacy and predictive biomarker of pegylated recombinant arginase on hepatocellular carcinoma. Invest New Drugs. (2021) 39:1375–82. doi: 10.1007/s10637-021-01111-8 PMC8426309 33856599

[208] Fang Y Liu W Tang Z Ji X Zhou Y Song S. Monocarboxylate transporter 4 inhibition potentiates hepatocellular carcinoma immunotherapy through enhancing T cell infiltration and immune attack. Hepatology. (2023) 77(1):248–62. doi: 10.1002/hep.32348 35043976

[209] Wang X Zhang H Yang H Bai M Ning T Deng T. Exosome-delivered circRNA promotes glycolysis to induce chemoresistance through the miR-122-PKM2 axis in colorectal cancer. Mol Oncol. (2020) 14:539–55. doi: 10.1002/1878-0261.12629 PMC7053238 31901148

[210] Wang C Huang X Wu Y Wang J Li F Guo G. Tumor cell-associated exosomes robustly elicit anti-tumor immune responses through modulating dendritic cell vaccines in lung tumor. Int J Biol Sci. (2020) 16:633–43. doi: 10.7150/ijbs.38414 PMC6990923 32025211

[211] Chinnappan M Srivastava A Amreddy N Razaq M Pareek V Ahmed R. Exosomes as drug delivery vehicle and contributor of resistance to anticancer drugs. Cancer Lett. (2020) 486:18–28. doi: 10.1016/j.canlet.2020.05.004 32439419 PMC7327711

[212] Yang X Yang C Zhang S Geng H Zhu AX Bernards R. Precision treatment in advanced hepatocellular carcinoma. Cancer Cell. (2024) 42:180–97. doi: 10.1016/j.ccell.2024.01.007 38350421

[213] Wang Q Liu J Chen Z Zheng J Wang Y Dong J. Targeting metabolic reprogramming in hepatocellular carcinoma to overcome therapeutic resistance: A comprehensive review. BioMed Pharmacother. (2024) 170:116021. doi: 10.1016/j.biopha.2023.116021 38128187

